# Functional characterization of the *Ucp1*-associated oxidative phenotype of human epicardial adipose tissue

**DOI:** 10.1038/s41598-017-15501-7

**Published:** 2017-11-14

**Authors:** Kanta Chechi, Pierre Voisine, Patrick Mathieu, Mathieu Laplante, Sébastian Bonnet, Frédéric Picard, Philippe Joubert, Denis Richard

**Affiliations:** 1 Institut universitaire de cardiologie et de pneumologie de Québec (IUCPQ), 2725 Chemin Sainte-Foy, Québec, G1V 4G5 Canada; 2 Department of Medicine, Faculty of Medicine, Laval University, Quebec, QC Canada; 3 Department of Cardiovascular Surgery, Faculty of Medicine, Laval University, Quebec, QC Canada; 4 Laboratory of Cardiovascular Pathobiology, Department of Surgery, Faculty of Medicine, Laval University, Quebec, QC Canada; 5 Pulmonary Hypertension and Vascular Biology Research Group, Faculty of Medicine, Laval University, Quebec, QC Canada; 6 Department of Pharmacy, Faculty of Medicine, Laval University, Quebec, QC Canada; 7 Department of Anatomo-Pathology, Faculty of Medicine, Laval University, Quebec, QC Canada

## Abstract

Brown fat presence and metabolic activity has been associated with lower body mass index, higher insulin sensitivity and better cardiometabolic profile in humans. We, and others, have previously reported the presence of *Ucp1*, a marker of brown adipocytes, in human epicardial adipose tissue (eAT). Characterization of the metabolic activity and associated physiological relevance of *Ucp1* within eAT, however, is still awaited. Here, we validate the presence of *Ucp1* within human eAT and its ‘beige’ nature. Using *in-vitro* analytical approaches, we further characterize its thermogenic potential and demonstrate that human eAT is capable of undergoing enhanced uncoupling respiration upon stimulation. Direct biopsy gene expression analysis reveals a negative association between thermogenic markers and oxidative stress-related genes in this depot. Consistently, isoproterenol (Iso) stimulation of eAT leads to a downregulation of secreted proteins included in the GO terms ‘cell redox homeostasis’ and ‘protein folding’. In addition, cardiac endothelial cells exhibit a downregulation in the expression of adhesion markers upon treatment with Iso-stimulated eAT derived conditioned media. Overall, these observations suggest that *Ucp1*- associated metabolic activity plays a significant role in local tissue homeostasis within eAT and can plausibly alter its communication with neighboring cells of the cardiovascular system.

## Introduction

Increased eAT mass around the heart is a well-known risk factor for the development of coronary artery disease (CAD)^1^, cardiovascular disease^2^, heart failure and atrial fibrillation^3^ in humans. While eAT mass shares strong associations with visceral fat accumulation^4,5^, a strong relationship between eAT mass and development of CAD has also been reported independent of body mass index and other measures of body adiposity in multiple cohorts^6–8^. Epicardial adipocytes are uniquely positioned to influence the microenvironment of myocardium and coronaries, as they are not separated from the underlying myocardium with any fascia-like structure anatomically^9–13^. Therapeutic targeting of eAT to modulate its phenotype and associated cardiovascular outcomes has thus been proposed repeatedly^5,7,10^.

Human eAT is known to express *Ucp1*, a thermogenic protein uniquely found in brown adipocytes^14^. However, the functional relevance of *Ucp1* within eAT remains largely unknown. We have also reported the presence of *Ucp1* mRNA in human eAT previously, where *Ucp1* further exhibited direct association with circulating HDL-cholesterol levels in a cohort of patients with CAD^15^. In addition, most genes involved in thermogenesis and lipid metabolism shared an inverse relationship with the circulating TG levels^15^. To us, the relevance of these observations was two fold; one being the hypothetical relationship between the thermogenic phenotype of eAT as a marker of the total brown fat in the body with circulating lipid levels at a systemic level, and other being a plausible functional role for the thermogenic adipocytes of eAT at the local level. Thus, we sought to characterize the thermogenic phenotype and nature of eAT, as well as the physiological relevance of *Ucp1* within eAT in the context of its own biology and its interaction with cells of the cardiovascular system. We hypothesize that thermogenic phenotype of eAT is a key feature that regulates its physiological health, where loss of *Ucp1*-mediated metabolic activity associates with an increase in its mass and/or an adverse metabolic phenotype that further exacerbate the communication between eAT and cells of the cardiovascular system and *vice-versa* ^7^.

Here, we report that human eAT exhibits (i) a beige phenotype, (ii) significant thermogenic capacity and (iii) an ability to upregulate its uncoupling -machinery and -respiration upon stimulation. In addition, we demonstrate that thermogenic phenotype shares an inverse association with oxidative stress markers within eAT at the tissue level. Consistently, adrenergic stimulation of eAT leads to a down-regulation of secretory proteins involved in pathways related to cell redox homeostasis on one hand and alters its communication with cardiac endothelial cells on the other. These observations point towards a direct role for *Ucp1*-associated metabolic activity in the local regulation of tissue homeostasis within eAT that further influences its communication with other cells. Future work is needed to characterize the mechanisms that underlie our observations in greater detail.

## Results

### Human eAT has significant levels of *Ucp1* mRNA

**Figure 1:**
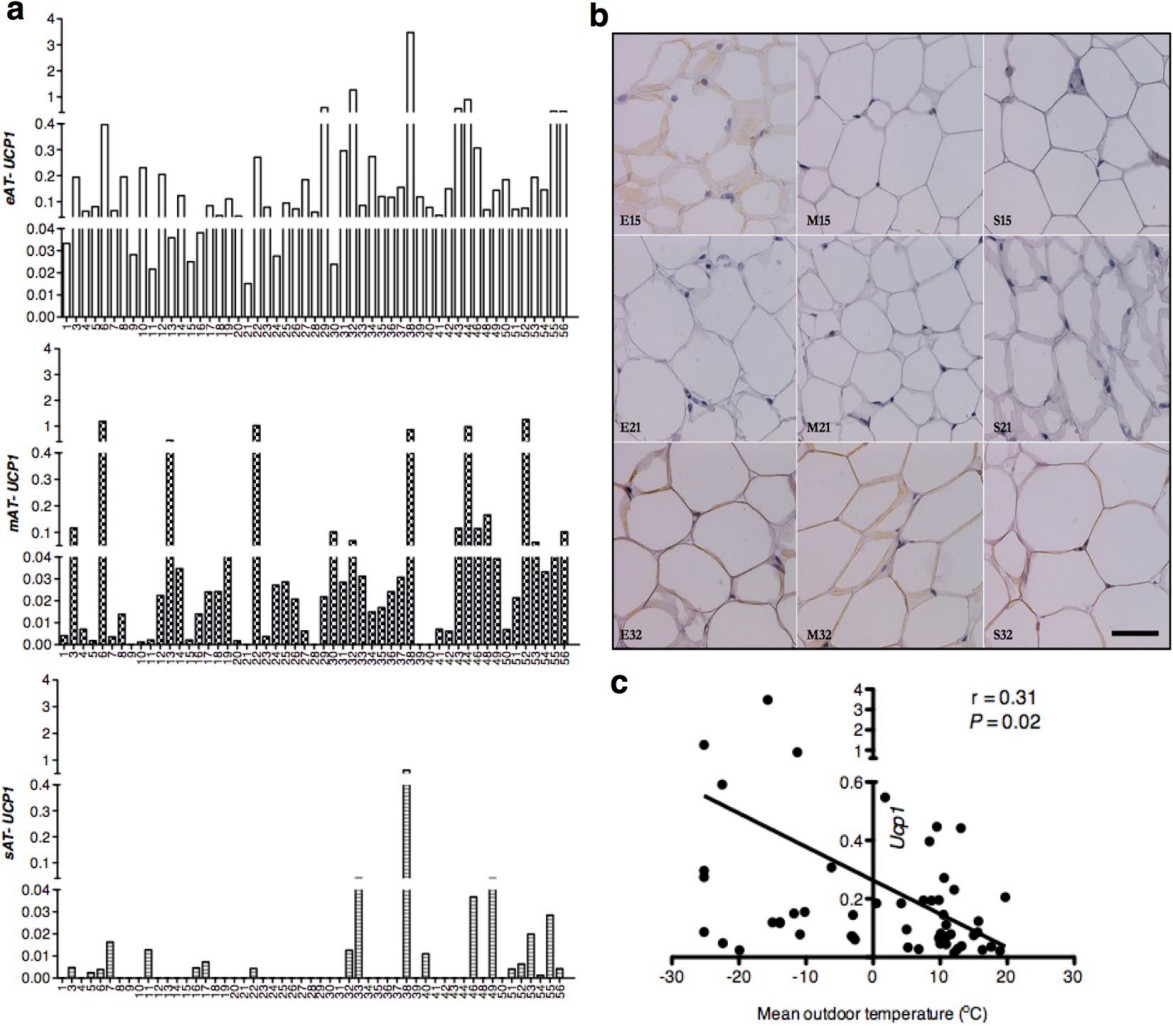
Assessment of *Ucp1* in eAT, mAT and sAT depots. *Ucp1* mRNA expression (**a**) in the eAT, mAT and sAT across individuals in our cohort (n = 53), and Ucp1-immunostaining (**b**) in eAT, mAT, sAT in selected patients (scale bar = 55 μm). Correlation analysis (**c**) between *Ucp1* mRNA in eAT with mean outdoor temperature on the day of tissue collection in our cohort (n = 53).

Human heart is surrounded by multiple layers of fat, which can be classified into epicardial *i*.*e*. fat lying inside the visceral pericardium, and paracardial or mediastinal adipose tissue (mAT) *i*.*e*. fat lying outside the parietal pericardium^16^. Unlike mediastinal adipocytes, epicardial adipocytes share a unique anatomic juxtaposition with the cardiomyocytes and coronaries; hence are of critical relevance to cardiac physiology. We^15^, and others^14^, have previously reported significant overexpression of *Ucp1* in human eAT relative to mAT and subcutaneous adipose tissue (sAT). Here, we repeated our previous observations in paired eAT, mAT and sAT biopsies (Table 1) obtained from a cohort of 53 subjects undergoing open-heart surgeries (clinical and biochemical details of the subjects given as Supplementary Table S1). However, a closer observation revealed that mAT also had variable yet detectable presence of *Ucp1* across individuals with few patients (*i*.*e*. H6, H13 and H22) expressing even higher *Ucp1* in mAT than eAT (Fig. 1a). In addition, mAT had higher levels of *Ucp1* relative to sAT (*P* ≤ 0.05) (Table 1). Clearly, sAT largely exhibited near negligible levels of *Ucp1* with few exceptions (Fig. 1a). Due to limited sample availability, immuno-histochemical approach was utilized to assess the Ucp1 protein levels in these patients. While no multilocular cells were observed, Ucp1 labeling was visible in an heterogeneous manner that was consistent with *Ucp1* mRNA levels across various fat depots of randomly selected individuals (Fig. 1b). We^17^, and others^18^, have previously reported that outdoor temperature can alter brown fat presence and activity in humans. In order to address whether *Ucp1* levels in eAT, mAT and sAT could be altered by environmental conditions, we looked for relationships between *Ucp1* and outdoor temperature as well as daylight in our cohort. Interestingly, we observed a significant negative correlation (*P* ≤ 0.05) between mean outdoor temperature on the day of tissue collection with *Ucp1* levels in eAT (Fig. 1c) but not in mAT or sAT. In contrast, no relationship was observed between daylength and *Ucp1* levels in either of these fat depots in our cohort (data not shown).

**Table 1:** Gene expression of individual genes in various categories in eAT, mAT and sAT biopsies.

	eAT (mean ± SEM)	mAT (mean ± SEM)	sAT (mean ± SEM)
***Thermogenic***			
*Ucp1*	0.248 ± 0.070^a^	0.137 ± 0.043^b^	0.017 ± 0.011^c^
*Ppargc1a*	0.089 ± 0.007^b^	0.180 ± 0.017^a^	0.102 ± 0.117^b^
*Prdm16*	0.226 ± 0.019^a^	0.295 ± 0.317^a^	0.157 ± 0.014^b^
*Cpt1b*	2.070 ± 0.262^a^	2.693 ± 0.325^a^	0.837 ± 0.107^b^
*Cox4i1*	0.843 ± 0.038	0.950 ± 0.054	0.979 ± 0.095
***Immune***			
*Il6*	0.071 ± 0.033^a^	0.052 ± 0.017^ab^	0.046 ± 0.018^b^
*Tnf*	0.020 ± 0.003a	0.024 ± 0.004a	0.007 ± 0.001b
*Ccl2*	3.759 ± 0.682	8.166 ± 2.993	4.570 ± 1.025
*Cd68*	0.467 ± 0.052^a^	0.625 ± 0.064^a^	0.306 ± 0.054^b^
*Mrc1*	5.119 ± 0.536^a^	5.902 ± 0.494^a^	3.622 ± 0.343^b^
*Ccl18*	0.200 ± 0.035^a^	0.140 ± 0.027^a^	0.109 ± 0.041^b^
***Extracellular matrix***			
*Timp1*	0.336 ± 0.052^a^	0.108 ± 0.009^b^	0.0705 ± 0.008^c^
*Mmp9*	2.777 ± 0.450	5.099 ± 1.547	5.189 ± 3.086
*Col3a1*	0.227 ± 0.016^c^	0.364 ± 0.033^b^	0.564 ± 0.051^a^
*Col6a3*	0.494 ± 0.047	0.519 ± 0.044	0.516 ± 0.052
***Oxidative stress***			
*Cyba*	2.097 ± 0.190^a^	2.180 ± 0.191^a^	1.136 ± 0.094^b^
*Hif1a*	0.390 ± 0.034^a^	0.443 ± 0.042^a^	0.211 ± 0.024^b^
*Tgfb1*	0.755 ± 0.075^ab^	0.793 ± 0.069^a^	0.428 ± 0.036^b^
*Ncf1*	0.107 ± 0.028^a^	0.084 ± 0.025^a^	0.012 ± 0.002^b^
***Adipose tissue growth and function***			
*Slc2a4*	2.949 ± 0.400^a^	3.188 ± 0.557^a^	1.409 ± 0.278^b^
*Cebpa*	11.670 ± 1.213^a^	10.180 ± 1.314^a^	5.994 ± 0.629^b^
*Adipoq*	2.786 ± 0.284	2.724 ± 0.357	2.088 ± 0.213
*Fabp4*	1.622 ± 0.089^c^	2.912 ± 0.181^b^	3.809 ± 0.193^a^
*Pparg*	2.333 ± 0.151^a^	1.902 ± 0.172^ab^	1.866 ± 0.264^b^
***Adrenergic receptors***			
*Adrb1*	5.067 ± 0.573^a^	6.539 ± 1.043^a^	2.455 ± 0.429^b^
*Adrb2*	1.094 ± 0.084	1.447 ± 0.172	2.038 ± 0.533
*Adrb3*	0.023 ± 0.004	0.029 ± 0.006	0.020 ± 0.004
*Adra2a*	1.767 ± 0.125^b^	3.565 ± 0.416^a^	4.458 ± 0.608^a^
*Adrb1/2a*	2.853 ± 0.230^a^	1.882 ± 0.239^b^	1.463 ± 0.419^c^
*Adrb2/2a*	0.683 ± 0.045^a^	0.484 ± 0.058^b^	0.316 ± 0.034^c^
*Adrb3/2a*	0.016 ± 0.003	0.012 ± 0.003	0.015 ± 0.004

Data are expressed as mean ± SEM, n = 53. Randomized block ANOVA was performed on the log-transformed data using R. Superscripts represent ^a^ *P* ≤ 0.05.

Similar to *Ucp1*, other thermogenic markers individually (*i*.*e*. *Prdm16* and *Cpt1b*) (Table 1), and as a group (*Ucp1*, *Ppargc1a*, *Prdm16*, *Cpt1b*, *Cox4i1*) (Table 2) were upregulated in both eAT and mAT relative to sAT (*P* ≤ 0.05).

**Table 2:** Expression of genes grouped in various categories in eAT, mAT and sAT biopsies.

	eAT	mAT	sAT
Thermogenesis	−1.10 ± 0.10^a^	−1.08 ± 0.10^a^	−2.07 ± 0.10^b***^
Beige	−0.57 ± 0.16^a^	−1.11 ± 0.16^b*^	−2.22 ± 0.16^c***^
White	−1.15 ± 0.09^c***^	0.11 ± 0.09^b^	0.51 ± 0.09^a^
Immune	−1.68 ± 0.12^a^	−1.82 ± 0.12^a^	−2.59 ± 0.12^b***^
Extracellular matrix	−1.00 ± 0.08	−1.06 ± 0.08	−1.12 ± 0.08
Oxidative stress	−1.15 ± 0.12^a^	−1.07 ± 0.12^a^	−2.03 ± 0.12^b***^
AT growth & function	1.08 ± 0.11^a**^	0.88 ± 0.11^a*^	0.52 ± 0.11^b^

Data are expressed as log of least square means ± SE, n = 53. Genes were grouped in various categories as the following: *Ucp1*, *Ppargc1a*, *Prdm16*, *Cpt1b*, *Cox4i1* as ‘thermogenesis’-related genes; *Tbx1*, *Tmem26*, *Tnfrsf9*, *P2rx5*, *Slc36a2* as ‘beige’-related genes’; *Shox2*, *Hoxc9*, *Slc7a10*, *Lep* as ‘white’-related genes; *Il6*, *Ccl2*, *Tnf*, *Ccl18*, *Mrc1*, *Cd68* as ‘immune’-related genes; *Col6a3*, *Col3a1*, *Mmp9*, *Timp1* as ‘extracellular matrix’-related genes; *Hif1a*, *Ncf1*, *Cyba*, *Tgfb1* as ‘oxidative stress’-related genes; *Slc2a4*, *Adipoq*, *Pparg*, *Fabp4*, *Cebpa* as the ‘AT growth and function’-related genes. Groups were compared using a multivariate randomized block ANOVA model. Superscripts represent significance of *P* ≤ 0.05. *Represents P ≤ 0.05, **represents P ≤ 0.01, ***represents P ≤ 0.001. AT: Adipose tissue.

### Human eAT is beige in nature

**Figure 2:**
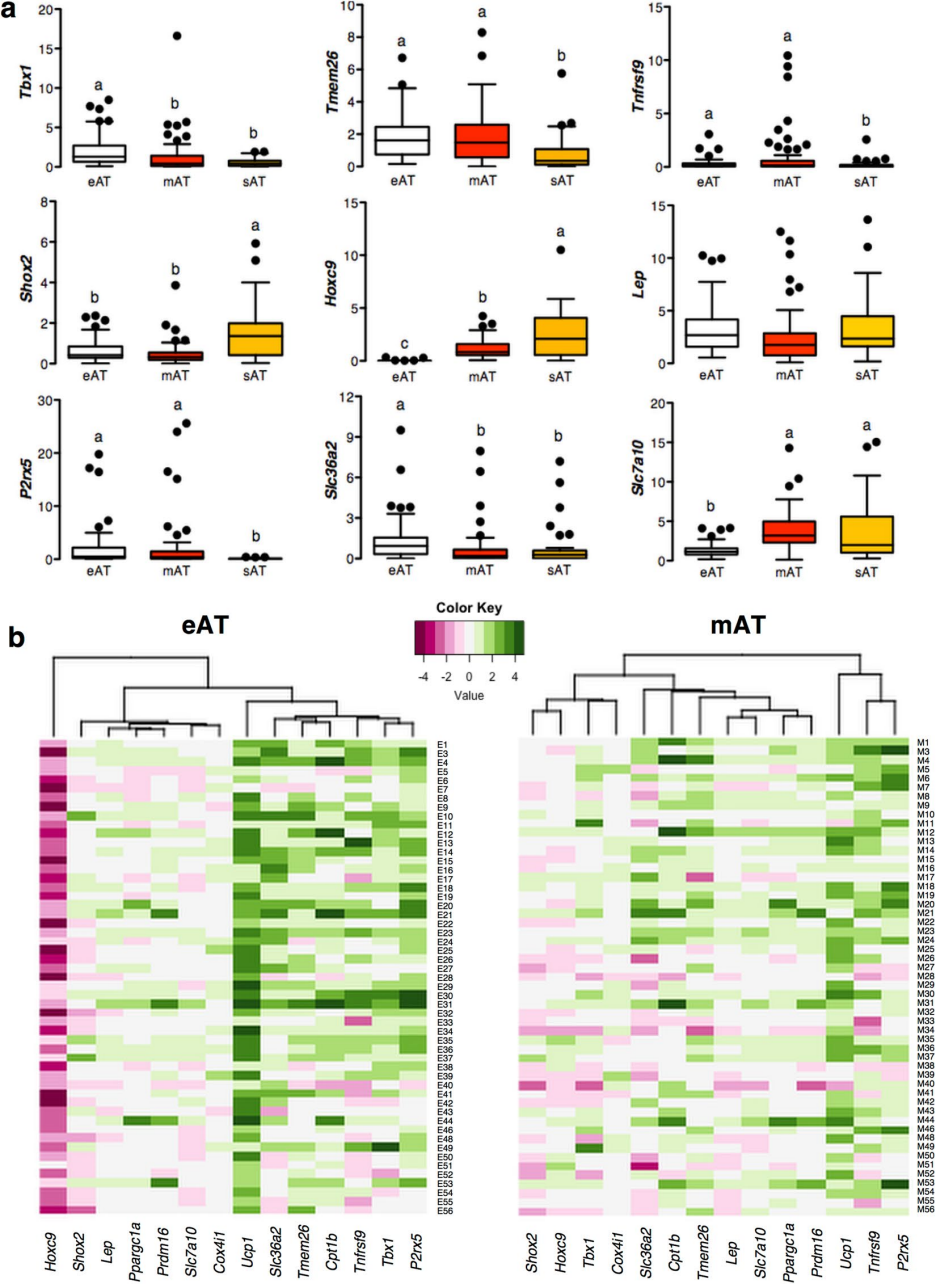
Assessment of the nature of eAT, mAT and sAT depots. Expression of beige (*Tmem26*, *Tbx1*, *Tnfrsf9*, *Slc36a2*, *P2rx5*) and white fat (*Shox2*, *Hoxc9*, *Slc7a10*, *Lep*) markers (**a**) in the eAT, mAT and sAT biopsies of the main cohort (n = 53). Data are expressed as mean ± SEM. Differences were estimated using randomized block ANOVA on log-transformed data. Different alphabets represent *P* ≤ 0.05. Heatmaps (**b**) representing the cluster analysis of thermogenic-, beige- and white-fat marker genes in eAT and mAT depots when data are expressed as log-fold change relative to sAT (n = 53). Each column contains the data from a specific gene, and each row contains data from single patient. Green color represents overexpression- whereas maroon color represents lower expression- of a specific gene in eAT or mAT relative to sAT. The dendrogram shows the degree of correlation of the genes as assessed by hierarchical clustering.

Owing to the recent developments in brown fat biology, *Ucp1*-positive adipose depots are now classified as being either classic brown or beige in nature. Distinction lies in the anatomic location, developmental ontogeny and molecular profiling of these depots^19,20^. In order to assess the nature of human eAT, we documented the expression of previously established^19,21–23^ key gene markers that can label classic brown, beige and white fat depots. Our objectives were two-fold; to assess the nature of eAT, and to determine whether these markers have any physiological relevance and hence associate with patient characteristics such as obesity, diabetes and CAD. While classic brown fat markers *Zic1* and *Lhx8* were not detectable in any of the fat depots, beige markers *Tbx1* and *Slc36a2* were specifically upregulated in eAT (*P* ≤ 0.05), whereas *Tmem26*, *P2rx5* and *Tnfrsf9* were overexpressed in both eAT and mAT relative to sAT (*P* ≤ 0.05) (Fig. 2a). Similarly, while eAT had lowest level of white fat markers *Hoxc9* and *Slc7a10* expression (*P* ≤ 0.001), *Shox2* was downregulated in both eAT and mAT relative to sAT (*P* ≤ 0.05) (Fig. 2a). Thus, both eAT and mAT exhibited beige fat-associated gene expression pattern.

To clarify the differences in the nature of eAT and mAT, hierarchical cluster analysis was performed on the log ratios of each of these gene markers in eAT and mAT relative to sAT. Noteworthy, eAT had a much higher fold upregulation in *Ucp1* and downregulation in *Hoxc9* expression relative to sAT (Fig. 2b). In addition, all known beige markers including *Tmem26*, *Slc36a2*, *Tnfrsf9*, *Tbx1* and *P2rx5* clustered with *Ucp1* only in eAT but not in mAT (Fig. 2b). Consistently, when grouped together, eAT had highest levels of beige marker expression and lowest levels of white fat marker expression (*P* ≤ 0.001), whereas mAT had an expression pattern that was intermediate of eAT and sAT (*P* ≤ 0.001) (Table 2). These data indicate that different fat depots even within close anatomic proximity can exhibit variable degrees of beige and white phenotypes in human body.

Gene markers included in the current study are usually investigated to label a fat depot as classic brown-, beige- or white fat depot, however their functional relevance, if any, remains obscure to-date. In order to address their depot-specific physiological relevance, we assessed whether expression of these genes both individually or as a group was affected by obesity, diabetes and CAD in eAT, mAT and sAT. No differences were observed in any of these markers as a group in any of the studied depots, except for a trend (*P* = 0.083) for reduction in beige marker expression in the eAT of patients with CAD (Supplementary Table S3b), and a significant down-regulation of beige markers in mAT of diabetic patients (*P* ≤ 0.001) (Supplementary Table S4b). However, when assessed individually, *Lep* expression was increased in both eAT and mAT, but not sAT, of obese individuals relative to lean (*P* ≤ 0.05), whereas beige fat marker *Slc36a2* expression was reduced in overweight individuals relative to lean (*P* ≤ 0.05) in eAT alone (Supplementary Table S2c). Similarly, white fat marker *Hoxc9* expression in sAT was reduced in overweight relative to lean individuals (*P* ≤ 0.05) (Supplementary Table S2c). Presence of CAD was associated with a down-regulation of beige marker *Tmem26* in eAT (*P* ≤ 0.05) and of *Cpt1b* and *Prdm16* in sAT (*P* ≤ 0.05) (Supplementary Table S3c). Similar to obesity, diabetes was associated with an increase in *Lep* expression in eAT (*P* ≤ 0.05), but a downregulation of *Slc36a2* expression in mAT (*P* ≤ 0.05) (Supplementary Table S4c). Overall, these data point towards depot-specific functional relevance for each of these markers and/or disease-specific mechanistic variations among eAT, mAT and sAT.

### Human eAT can upregulate thermogenic phenotype and uncoupled respiration upon stimulation

In order to address the question of whether presence of *Ucp1* in eAT translates into any metabolic activity and whether eAT retains the ability to upregulate its thermogenic machinery upon stimulation, eAT, mAT and sAT biopsies were subjected to primary cell culture. Preliminary assessment of *in-vitro* differentiated adipocytes derived from 4 different patients subjected to oxygen consumption rate (OCR) analysis revealed three key observations (Supplementary Figure 1a,b). First, differentiation variability among samples and tissue types, where sAT-derived adipocytes consistently exhibited highest level of differentiation (Supplementary Figure 1a). Second, basal OCR levels reflected the state of differentiation level for each cell type (Supplementary Figure 1a,b). Third, despite enhanced differentiation, sAT adipocytes consistently exhibited a drop in OCR upon addition of oligomycin, an ATP-synthase inhibitor, which was not the case for eAT and mAT adipocytes (Supplementary Figure 1a). This would point towards a stronger reliance on ATP synthase for respiration in sAT-derived adipocytes, whereas eAT- and mAT- derived adipocytes rely more upon uncoupled respiration. It is also important to note that when a similar level of differentiation was achieved (for *e*.*g*. mAT and sAT in subject 4), mAT adipocytes had higher levels of basal respiration, no response to oligomycin and highest levels of FCCP-stimulated respiration, all of the hallmark features of an OCR profile of a thermogenic adipocyte (Supplementary Figure 1b).

**Figure 3:**
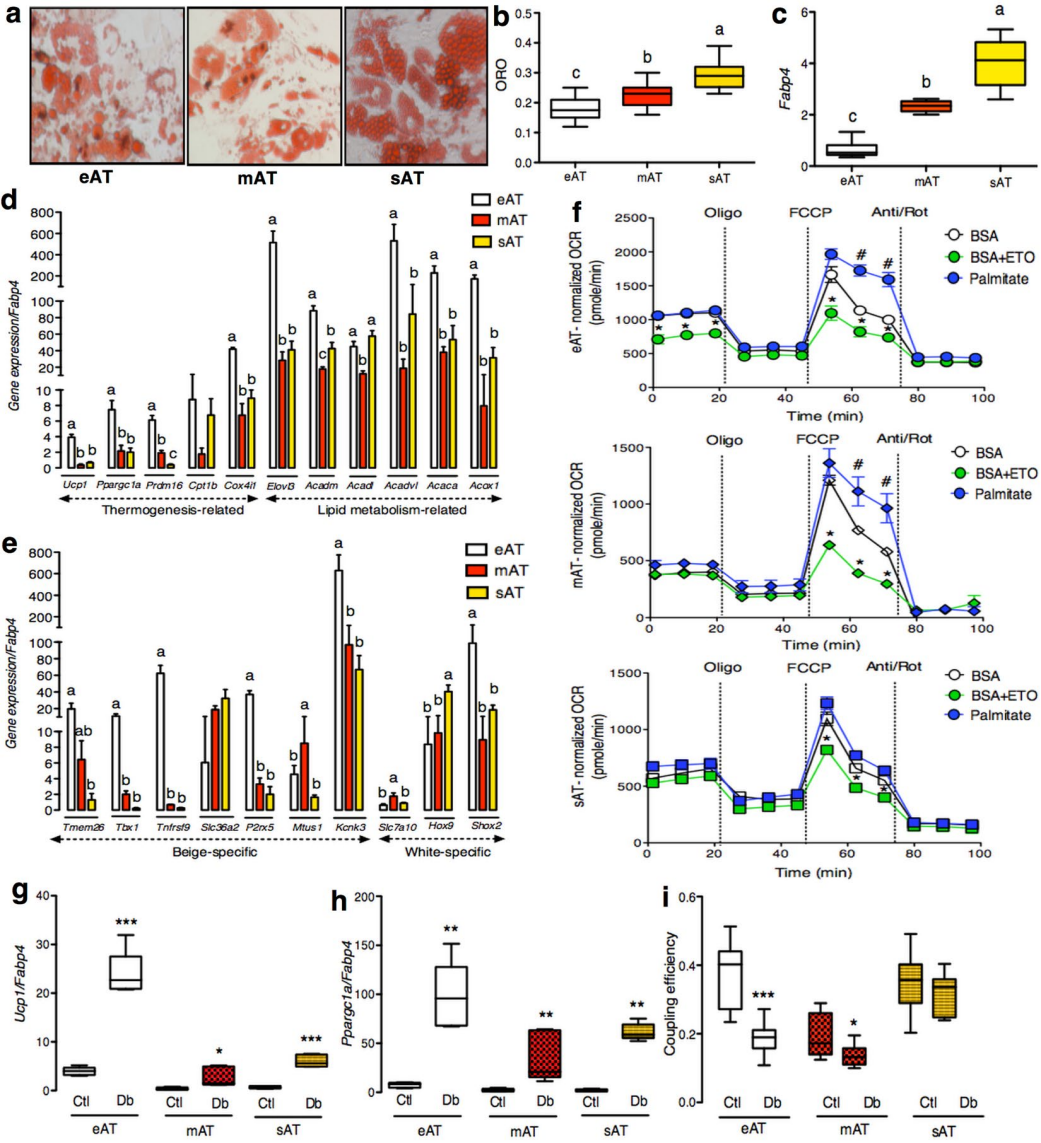
*In-vitro* characterization of eAT-, mAT- and sAT-derived adipocytes. ORO staining (**a**), ORO quantification (n = 10) (**b**), mRNA expression of *Fabp4* (n = 5) (**c**), thermogenesis and lipid metabolism-related gene markers (n = 5) (**d**) and beige-, white- fat related gene markers (n = 5) (**e**) in the *in-vitro* differentiated eAT-, mAT- and sAT- adipocytes. (**f**) FAO analysis using OCR assays in eAT-, mAT- and sAT- derived adipocytes (n = 5). Gene expression of *Ucp1* (n = 5) (**g**), *Ppargc1a* (n = 5) (**h**) and coupling efficiency (n = 10) (i) of eAT-, mAT- and sAT-derived adipocytes with- or without- pretreatment with dibutyryl-cAMP (Db). The eAT-, mAT- and sAT-derived adipocytes were obtained after pooling samples from 9 patients. Data represents mean ± SEM. Significance of difference was determined by one-way ANOVA followed by Tukey’s *post hoc* analysis or unpaired t-tests as needed. Different alphabets represent *P* ≤ 0.05. *Represents *P* ≤ 0.05, **represents *P* ≤ 0.01, ***represents *P* ≤ 0.001. For FAO, ^#^represents *P* ≤ 0.05 between palmitate and BSA and *represents *P* ≤ 0.05 between BSA+ETO and BSA. ORO, oil red O; FAO, fatty acid oxidation; OCR, oxygen consumption rate; BSA, bovine serum albumin; ETO, etomoxir.

Due to limited sample availability and shorter life span of eAT-derived primary adipocytes, preadipocytes from 9 patients were pooled to generate enough working material to address our specific questions. To begin with, eAT adipocytes had lowest level of differentiation followed by mAT and sAT, evident from neutral lipid staining (ORO) and *Fabp4* mRNA levels (*P* ≤ 0.05) (Fig. 3a,b,c), which were also used for normalizing the OCR and gene expression data, respectively. eAT adipocytes had higher expression of key thermogenic markers *Ucp1*, *Ppargc1a*, *Prdm16* and *Cox4i1* (*P* ≤ 0.05) than sAT at the basal level (Fig. 3d). eAT adipocytes also retained their beige phenotype in culture indicated by higher expression of beige markers *Tbx1*, *Tnfrsf9*, *P2rx5*, *Kcnk3* and lower expression of *Hoxc9* relative to sAT (*P* ≤ 0.05) (Fig. 3e). In contrast, mAT adipocytes exhibited higher expression of recently identified human brown fat marker *Mtus1* ^23^ and white fat marker *Slc7a10* along with lower expression of *Hoxc9* relative to sAT (*P* ≤ 0.05) (Fig. 3e). Overall, these adipocytes retained a gene expression pattern that was consistent with our observations made in tissue biopsies, where eAT largely exhibited a beige phenotype and sAT a white phenotype whereas mAT showed a pattern intermediary of eAT and sAT (Fig. 2a,b). OCR profile of these adipocytes under non-stimulated conditions further revealed higher maximal respiration and spare respiratory capacity in eAT and mAT relative to sAT, pointing towards the fact that both eAT and mAT adipocytes possess a higher capacity for respiration under stressed conditions (Supplementary Figure 2).

Lipid metabolism, both synthesis and oxidation, plays a significant role in the execution of thermogenesis. In addition, it has been suggested that eAT has higher level of lipid metabolism relative to other visceral fat depots^24,25^, however this has not been explored in human eAT beyond gene expression level so far. Thus, we sought to assess the state of lipid metabolism in these adipocytes. A number of key genes involved in lipid synthesis as well as oxidation such as *Elovl3*, *Acaca*, *Acadm*, *Acox1* and *Acadvl* were all upregulated in eAT relative to both mAT and sAT (*P* ≤ 0.05) (Fig. 3d). We further explored the parameters associated with fatty acid oxidation in these adipocytes using OCR analysis. The addition of palmitate led to significantly higher maximal respiration rates in both eAT and mAT but not in sAT adipocytes (*P* ≤ 0.05) (Fig. 3f). The addition of etomoxir, a fatty acid oxidation inhibitor, led to significant blunting of maximal respiration rates in all three cell types, indicating that regardless of their origin (*i*.*e*. eAT, mAT or sAT) adipocytes rely upon intracellular fat oxidation to support respiration under stressed conditions. Interestingly however, etomoxir addition was also associated with specific blunting of basal respiration in eAT adipocytes alone (*P* ≤ 0.05) (Fig. 3f). This observation points towards a unique reliance upon the intracellular lipids for basal respiration in eAT adipocytes, a distinctive feature of fat cells with thermogenic activity^26,27^.

To further buttress the thermogenic role of eAT adipocytes, we next tested their thermogenic reserve upon stimulation. While four- hour pretreatment with a cAMP analog, di-butyryl cAMP, was associated with an upregulation in *Ucp1* and *Ppargc1a* mRNA levels in each cell type (Fig. 3g,h), significant reduction in coupling efficiency was observed only for eAT and mAT (Fig. 3i). Of note, despite the presence of *Ucp1* in sAT derived adipocytes, no shift in their coupling efficiency was observed upon stimulation (Fig. 3i). Overall, eAT exhibited a higher thermogenic capacity at the basal level and also exhibited stronger upregulation in *Ucp1* mRNA and uncoupled respiration upon stimulation, indicating that *Ucp1* in eAT adipocytes is thermogenically competent.

### Thermogenic phenotype inversely associates with the oxidative stress markers in human eAT

Having established the thermogenic capabilities of eAT, we sought to assess the physiological relevance of the thermogenic phenotype in each of these fat depots. Although the primary goal of thermogenesis in the context of a classic brown adipose tissue is to generate heat to support the survival of a cold-exposed organism, the physiological relevance of a beige adipose tissue, such as the eAT, is largely unknown. We hypothesized that the thermogenic capacity and associated metabolic activity of *Ucp1* within eAT affects local tissue homeostasis. *Ucp1* within eAT may modulate its inflammation and oxidative stress levels and/or alter the capacity of eAT to expand. It may further alter the communication between eAT and its neighboring cells and tissues such as the coronary arteries.

In order to test our hypothesis, we first assessed the state of inflammation-, fibrosis-, oxidative stress- and adipose tissue growth and function-related gene expression both individually (Table 1) and as a group (Table 2) in eAT, mAT and sAT at the tissue level (n = 53). Most genes involved in inflammation such as *Tnf*, *Cd68*, *Mrc1* and *Ccl18* were upregulated in both eAT and mAT relative to sAT (*P* ≤ 0.05) individually (Table 1) and as a group (*P* ≤ 0.0001) (Table 2). Extracellular matrix markers as a group were not differentially expressed among various fat depots (Table 2). However, individually, eAT had highest expression of *Timp1* and lowest expression of *Col3a1* (*P* ≤ 0.05) relative to mAT and sAT (Table 1) whereas mAT exhibited intermediate levels of expression between eAT and sAT (*P* ≤ 0.05) (Table 1). Oxidative stress markers *Cyba*, *Hif1a* and *Ncf1* were upregulated in eAT and mAT than sAT (*P* ≤ 0.05) both individually (Table 1) and as a group (*P* ≤ 0.0001) (Table 2). Similarly, as a group, adipose tissue growth and function related genes were overexpressed in eAT and mAT than sAT (*P* ≤ 0.0001) (Table 2). Whereas, individually, *Slc2a4* and *Cebpa* were upregulated in eAT and mAT than sAT (*P* ≤ 0.05) (Table 1), whereas *Fabp4* was particularly downregulated in eAT (*P* ≤ 0.05) (Table 1). Overall, eAT and mAT exhibited higher levels of inflammation, oxidative stress and adipose tissue expansion related markers than sAT in our cohort.

In order to assess whether *Ucp1* presence within eAT and/or its beige nature has any impact on local tissue homeostasis, we sought to assess the relationship among thermogenic-, beige- and white fat- markers with each of the functional categories of genes described above. Principal component analysis followed by stepwise regression analysis for each group of genes revealed that thermogenic group associates negatively with oxidative stress markers in both eAT (*P* ≤ 0.01) and mAT (*P* ≤ 0.05) (Table 3). In fact, oxidative stress was the only significant contributor to the variance observed in the thermogenic phenotype of eAT (Table 3). In sAT, thermogenic group (without *Ucp1*) exhibited positive associations with immume- (*P* ≤ 0.001) and adipose tissue growth and function- related markers (*P* ≤ 0.001) (Table 3). In contrast to the thermogenic markers, beige group exhibited positive associations with the oxidative stress markers (*P* ≤ 0.001) in eAT and mAT (Table 3). Interestingly, beige markers had least expression in sAT and no associations were observed for this depot (Table 3). White fat group had negative associations with adipose tissue growth and function related markers in both eAT (*P* ≤ 0.001) and mAT (*P* ≤ 0.01), but a positive association in sAT (*P* ≤ 0.001) (Table 3).

**Table 3:** Relationship among thermogenic-, beige- and white fat- marker genes with all other gene categories in eAT, mAT and sAT biopsies.

	Thermogenesis	Beige	White
*eAT*			
Thermogenesis			
Beige			
White			
ECM			
Immune		2.95 ± 1.14* (14.2%)	
Oxidative stress	−4.41 ± 1.40** (25.6%)	4.84 ± 1.0*** (49.70%)	
AT growth & function			−4.58 ± 0.11*** (45.7%)
Total variance	36.10%	63.90%	52.40%
*mAT*			
Thermogenesis		3.53 ± 1.05** (7.60%)	3.15 ± 1.30* (9.30%)
Beige	4.90 ± 1.43** (20%)		−4.67 ± 1.36** (23.3%)
White	3.46 ± 1.43* (8.60%)	−4.05 ± 1.16** (10%)	
ECM			
Immune			
Oxidative stress	−2.93 ± 1.31* (8.7%)	7.43 ± 1.00*** (47.50%)	2.73 ± 1.25* (9.80%)
AT growth & function	−3.92 ± 1.14** (16.6%)		−3.34 ± 1.12** (15.7%)
Total variance	54%	67.80%	58%
*sAT*			
Thermogenesis			
Beige			
White			
ECM			
Immune	4.34 ± 0.94*** (33.70%)		
Oxidative stress			
AT growth & function	4.92 ± 0.78*** (46.70%)		3.63 ± 0.89*** (45.60%)
Total variance	83.50%		55.40%

Values represent stepwise regression coefficient estimate ± SE (% variance for each category) for various functional gene categories for each fat depot, n = 53. Relationships among these groups of genes (categorized in Table 2) were assessed using principal component analysis followed by stepwise regression as described in the methods section. *Represents *P* ≤ 0.05, **represents *P* ≤ 0.01, ***represents *P* ≤ 0.001. ECM: extracellular matrix; AT: Adipose tissue.

### Adrenergic stimulation of human eAT downregulates secreted proteins related to redox homeostasis and alters its cross-talk with cardiac endothelial cells

Sympathetic nervous system *via* β3-adrenergic receptors is known to be a strong controller of the thermogenic activity of rodent brown fat^20^, whereas the adrenergic receptor distribution in human eAT is currently unknown. We, thus, sought to assess the expression of adrenergic receptors in our cohort. *Adrb1* was overexpressed in both eAT and mAT relative to sAT (*P* ≤ 0.05) whereas *Adra2a* levels were specifically downregulated in eAT (*P* ≤ 0.05) (Table 1). No differences were observed in the expression of *Adrb2* and *Adrb3*. Interestingly, when data were expressed as a ratio between *Adrb1–2–3* and *Adra2a*, both *Adrb1/2a* and *Adrb2/2a* were upregulated in eAT followed by mAT and sAT (*P* ≤ 0.05) (Table 1), suggesting that specific downregulation of inhibitory α-adrenergic receptor *Adra2a* in eAT might be a factor that predisposes this depot to a higher signaling *via* β1- and β2-adrenergic receptors. Moreover, *Adra2a* expression was also upregulated specifically in eAT under conditions of obesity and diabetes (Supplementary Tables S2c and S4c), pointing towards a key role for this receptor in modulating adrenergic responsiveness of human eAT.

**Figure 4:**
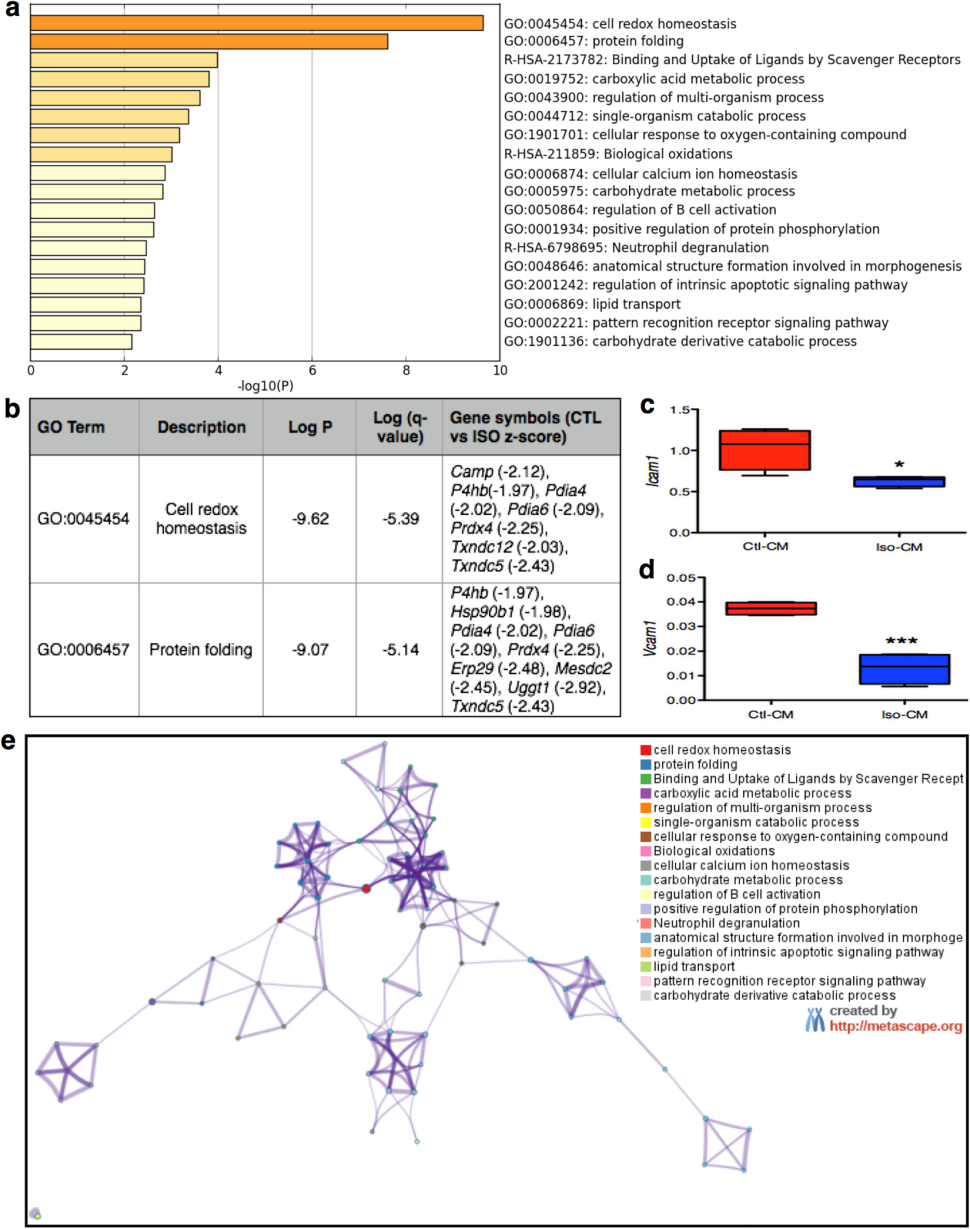
Analyzing the secretome of adrenergically stimulated eAT. Enrichment terms (**a**) identified by Metascape upon analysis of 48 secreted proteins that were differentially regulated in the Iso-CM relative to Ctl-CM. Description of the genes included in the two most significantly altered GO terms and their fold change in Iso-CM relative to Ctl-CM (**b**). Relationships among these enrichment terms displayed as a network (Metascape). Each term is represented by a circle node, where its size is proportional to the number of input genes falling into that term, and its color representing its cluster identity (*i*.*e*., nodes of the same color belong to the same cluster). Terms with a similarity score > 0.3 are linked by an edge (the thickness of the edge represents the similarity score). The network is visualized with Cytoscape (v3.1.2) with “force-directed” layout and with edge bundled for clarity. One term from each cluster is selected to have its term description shown as label (**e**). *Icam1* (**c**) and *Vcam1* (**d**) gene expression in primary cardiac endothelial cells upon treatment with Ctl- or Iso-CM (n = 4). Data represents mean ± SEM. Significance of difference was determined by unpaired t-tests. *Represents P ≤ 0.05, ***represents P ≤ 0.001. CM, conditioned media; Iso, isoproterenol; Ctl, control.

Next, we assessed whether adrenergic stimulation of eAT can alter its communication with neighboring cells. Conditioned media (CM) derived from control- and Iso- treated eAT explants were added to the primary human cardiac endothelial cell cultures for 24 hours. Out of the 4 patients tested, Iso-CM derived from one patient (subject 4) had significant down-regulation in the expression of adhesion markers *Icam1* and *Vcam1* in the endothelial cells relative to control-CM (*P* ≤ 0.05) (Fig. 4c,d). Considering that our adipose samples were derived from heart patients with multiple metabolic abnormalities, it was not surprising to see that all of the samples did not respond to Iso treatment similarly. Nonetheless, in order to assess the mechanisms that could explain the observations made in subject 4, we subjected respective Ctl and Iso-CM to liquid chromatography- mass spectrometric analysis. Comparative mass spectrometry analysis identified peptides that corresponded to 48 differentially expressed secreted proteins in the Iso-stimulated condition relative to control (Supplementary Table S6). GO and Metascape analyses of these 48 markers identified cell redox homeostasis and protein folding as two main biological processes with most significant log p-values (−9.62 and −9.07, respectively) (Fig. 4a,b). All of the genes included in these two GO terms were significantly downregulated upon Iso stimulation, thereby suggesting a downregulation of oxidative stress and associated mechanisms in human eAT upon adrenergic stimulation (Fig. 4b). Interestingly, all other biological processes that were identified with a significant p-value were closely interrelated (Fig. 4e) and largely reflected a change in the oxidative stress, inflammation and tissue metabolism related pathways (Fig. 4a,e). Detailed description of the genes associated with each of these pathways is given as Supplementary Table S7. Overall, these results indicate that adrenergic stimulation of eAT leads to a downregulation of oxidative stress as a primary mechanism, which plausibly further alters its communication with neighboring endothelial cells.

## Discussion

Presence of brown fat in the pericardial region was first reported by Juliet Heaton in 1979^28^, whereas the first demonstration of *Ucp1* mRNA expression in the human eAT came much later in 2009^14^. We also reported positive associations among the expression of thermogenic gene markers in human eAT and circulating HDL cholesterol levels in a previous study^15^. However, a direct investigation of the oxidative potential and physiological relevance of *Ucp1* in human eAT has not been undertaken so far. Considering that *Ucp1-*mediated uncoupling has been associated with enhanced energy expenditure and improved markers of metabolic health in rodents and humans^19,29–32^, we hypothesized a more direct role for *Ucp1* in regulating the fatty acid and metabolic homeostasis of eAT thereby serving as a key regulator of the relationship between eAT and CVD^7^. It is also important to highlight that eAT is a prototypical human brown fat depot, which clearly exhibits multilocular *Ucp1* expressing adipocytes in the neonatal period, and transitions into a white-like fat depot as we grow^33^. Studying eAT, therefore, further allows us to develop a better understanding of the physiological role of human brown fat.

Human eAT has also been the focus of significant clinical attention, where using various imaging modalities, multiple studies have indicated that an increase in eAT mass and volume associates with the incidence and occurrence of CAD^1,2^, and can even serve as a predictor of future coronary events independent of BMI^6,8,13,34,35^. However, a number of such studies do not segregate between eAT and the fat lying outside the pericardium *i*.*e*. paracardial or mediastinal fat, instead suggesting a role for pericardial fat (*i*.*e*. eAT and mAT combined) in the pathophysiology and prognosis of CAD^36–38^. Besides the differences in the anatomic location, eAT and mAT are also segregated developmentally, where eAT originates from mesothelial cells migrating from the septum transversum and mAT originates from primitive thoracic mesenchymal cells^39^. Unlike eAT, mAT also receives its blood supply from non-coronary sources^39^. Nevertheless, mAT has been reported to express *Ucp1* ^15,40,41^ and has also been recognized as a site for 18^F^-FDG uptake^42,43^, as a marker of brown fat activity, in humans. Thus, in the current study, we sought to characterize as well as compare the thermogenic phenotype of eAT and mAT in relation to sAT in a cohort of patients undergoing cardiac surgical procedures at our Institute.

To begin with, we validated previous observations of *Ucp1* overexpression in eAT relative to mAT and sAT^14,15^. Moreover, much like 18^F^-FDG uptake activity in the supraclavicular region^17,18^, we observed a significant negative association between *Ucp1* and mean outdoor temperature in eAT alone. While cumulatively, mAT exhibited much lower level of *Ucp1* expression relative to eAT, we could easily identify individuals that exhibited higher levels of *Ucp1* in mAT than eAT. What determines the *Ucp1* levels in each of these depots remains to be determined, however, it is clear that eAT exhibited consistent presence of *Ucp1* across all individuals despite the significant variability in the patient population. *Ucp1*-immunostaining further revealed that both eAT and mAT are largely unilocular white-like adipose depots that do not exhibit uniform *Ucp1* immuno-reactivity at the cellular level. Looking at their white fat-like phenotype, we hypothesized for both of these fat depots to be beige in nature and indeed the expression profiling of known molecular markers revealed eAT to exhibit a stronger beige phenotype than mAT. In contrast, mAT exhibited a stronger white-fat phenotype than eAT. Overall, we observed a gradient of beige to white phenotype from eAT to mAT to sAT in the human thoracic region. Such gradient of classic brown to beige to white phenotype has also been suggested to exist in the neck region in humans previously where as one moves from the center of neck towards the skin, the fat depots become increasingly white-like^22,44,45^. The question of whether the beige phenotype of eAT is derived from all its cells being beige adipocytes with a unilocular appearance or whether there are few unilocular- *Ucp1* expressing beige adipocytes interspersed between *Ucp1*-negative white adipocytes remains to be conclusively discerned.

Although eAT and mAT have previously been reported to exhibit a beige-like phenotype^41,46^, the previous studies did not investigate the entire range of markers studied here in addition to the direct comparison of the beige phenotype of eAT and mAT in a pairwise manner. As mentioned before, in assessing these markers, our objectives were two-fold; labeling of eAT, mAT and sAT on one hand and assessing the physiological relevance of these markers on the other. It is important to note that while some of commonly assessed beige markers (*Tmem26*, *Tnfrsf9*, *P2rx5*) were upregulated in both eAT and mAT, others (*Tbx1*, *Slc36a2*, *Hoxc9*) were differentially expressed in eAT alone. Noting that some of these adipocyte markers are known to be development-associated genes^47^, it is likely that these gene expression profiles simply point towards the distinctive developmental lineage of eAT and mAT depots. In assessing the physiological relevance of these markers, *Slc36a2*, a beige marker specifically upregulated in eAT and *Hoxc9*, a white fat marker specifically upregulated in sAT, were found to exhibit a differential gene expression in lean and overweight individuals (Supplementary Table 2b). Interestingly, their expression pattern is indicative of an adaptive phenomenon occurring during obesity where a reduction in the expression visible in overweight condition is not retained during obesity with expression levels being similar to lean condition. In contrast, presence of CAD was associated with a down-regulation of beige marker *Tmem26* in eAT alone. Differential regulation of these genes indeed points towards involvement of distinctive mechanisms at play during obesity and CAD. However, whether any of these genes have a direct functional relevance to the pathogenesis of these conditions in specific depots remains to be seen.

In order to make the case for a physiological role for *Ucp1*-associated metabolic activity within eAT, we tested its functional capability using an *in-vitro* approach. In particular, eAT-derived adipocytes exhibited significantly higher levels of most genes involved in thermogenesis and maintained a beige profile in culture. In addition, most genes involved in lipid-synthesis and -oxidation were upregulated in eAT-derived adipocytes that also exhibited a beta-oxidation profile consistent with a higher thermogenic capacity at the basal level. Importantly, eAT-derived adipocytes exhibited a unique reliance on intracellular lipid stores under basal conditions, which was not seen for other cell types. Intracellular lipid stores are known to be key players in the execution of thermogenesis in humans and rodents^26,27,32^, thus, this observation provides support for a plausible thermogenic role for eAT. Upon mimicking sympathetic stimulation, although all cell types responded with an upregulation of *Ucp1* mRNA expression, the reduction in coupling efficiency was only seen in eAT- and mAT- but not for sAT- derived adipocytes. Whether it is simply a matter of level of stimulation or presence of cell autonomous factor/mechanism that suppresses *Ucp1* activity in sAT remains to be determined. In this regard, we recently reported that despite the presence of *Ucp1* mRNA in inguinal fat depot, no metabolic activity was observed in this depot in rodents during chronic cold-exposure^48^. It is important to note that while *Ucp1* mRNA expression is often assessed to comment on the brown-like properties of human subcutaneous fat^49^, at least one study has reported functional thermogenesis in the *in-vitro* differentiated brown-like preadipocytes derived from human neck fat^50^. Fatty acids in the media have been shown to mimic *Ucp1*-mediated uncoupling and hence it was suggested that addition of BSA is critical to the OCR studies focused on assessing *Ucp1* activity^51^. Presence of BSA in our study may have been responsible for the difference in our observations relative to that of others. In addition, we acknowledge that occurrence of *Ucp1*-independent thermogenesis *via* creatine driven futile cycling has been reported in rodent beige fat during cold exposure^52^. While we did not directly assess the parameters associated with *Ucp1*-independent futile cycling pathways in the current study, their relevance to eAT mediated thermogenesis cannot be excluded and should be explored in the future studies.

Having established a clear thermogenic- phenotype and -potential of human eAT, we sought to assess the physiological relevance of *Ucp1* presence within eAT. We have previously reported that thermogenic markers in eAT associated negatively with circulating TG levels and positively with HDL-cholesterol levels at a systemic level in a separate cohort^15^. Interestingly, when assessed as a group, thermogenic gene markers exhibited a positive correlation with HDL-cholesterol and negative correlation with circulating TG levels in this cohort as well, thereby affirming our previous observations (data not shown). Next, we focused on the physiological relevance of thermogenic- and beige- phenotypes of eAT at a local level using a two-pronged approach. On one hand, we looked for relationships among thermogenic-, beige-, and white-fat marker groups with the presence of obesity, CAD and diabetes, and on the other, we assessed the relationship among these markers and multiple other markers of adipose tissue health such as growth and function, inflammation, oxidative stress and extracellular matrix components at the local level. We affirm previously reported observations that eAT and mAT exhibit an inflammatory profile relative to sAT^40,53,54^, however, a specific upregulation in inflammation relative to sAT in case of CAD reported previously^55^ was seen only for mAT (Supplementary Table 3b) but not for eAT in our cohort. None of the other family of genes exhibited any differential expression of genes under obesity, diabetes and CAD. Interestingly, however, despite being upregulated in eAT and mAT relative to sAT as a group (Table 2), thermogenic gene markers associated inversely with oxidative stress markers in both eAT and mAT but not sAT (Table 3). In contrast, and rather unexpectedly, beige markers as a group exhibited a positive association with oxidative stress markers in both eAT and mAT (Table 3). These observations highlight the disconnect between thermogenic, beige- and white- fat marker genes at the expression and functional level. Whilst thermogenic genes (especially *Ucp1*) are capable of modulating their-function and -activity at a certain level of expression, this is likely not the case for beige- and white- fat markers. It is interesting to note that while white-fat markers associated inversely with adipose tissue growth and function markers in eAT and mAT, this association was positive for sAT (Table 3). Importantly, the expression of white fat- specific markers was much lower in eAT and mAT than sAT (Table 2 and Fig. 2a), suggesting that plausibly as a beige fat depot whitens, its growth and function is negatively impacted. In contrast, white fat markers of a white fat depot likely point towards a healthy phenotype, evident from the observation that *Hoxc9* expression is reduced in overweight relative to lean individuals in the sAT depot (Supplementary Table 2b).

Next, we studied the AR distribution in human eAT. Unlike rodent brown and beige fat, human eAT seems to be regulated by *Adrb1* and *Adrb2* mediated sympathetic signaling, with *Adra2a* being a key player in the modulation of its adrenergic responsiveness. Whether adrenergic stimulation of eAT depot could alter its interaction with the cells of the cardiovascular system is currently unknown. Considering that endothelial cell dysfunction is a key aspect of atherosclerosis^56^ and adhesion molecules play a central role in the phenomenon^57^, we focused on the relationship between eAT and endothelial cell-activation. That eAT is capable of communicating with cardiomyocytes, endothelial cells and vascular smooth muscle cells has been demonstrated in multiple studies^58,59^, this relationship being negative where secretory products from eAT under conditions of diabetes and CAD were used^58,59^. In contrast, we observed that sympathetic stimulation of eAT was associated with a shift in its secretion profile that further associated with a downregulation in the expression of adhesion molecules *Vcam1* and *Icam1* in the human coronary artery endothelial cells. Although adrenergic agonism is a strong stimulator of thermogenic process and we observed an increase in the expression of *Ucp1* and other thermogenic genes in eAT explant upon Iso stimulation (data not shown), we acknowledge that our observations can not be exclusively attributed to thermogenic induction of eAT adipocytes alone. Instead, the observed differences may have their origin in shifts in the microenvironment mediated by direct effects of sympathetic stimulation on various cell types within eAT. Nonetheless, we observed a favorable shift in the relationship between eAT and endothelial cells post-sympathetic stimulation.

It is conceivable that changes in a specific factor within eAT secretome underlie our observations, however, we observed a statistically significant shift in 48 secreted proteins post-stimulation. Metascape and GO term analysis of these 48 proteins revealed cell-redox homeostasis and protein folding to be the most conspicuously shifted terms, most involved genes belonging to the category of protein disulfide isomerases showing a downregulation upon sympathetic stimulation (Fig. 4c). These observations were complimentary to the observation made with gene expression analyses of eAT biopsies, providing strength to the suggestion that *Ucp1* activity within eAT associates with a reduction in the oxidative/endoplasmic reticulum stress-related pathways in this depot. Associations between *Ucp1*-induction and -activity with mitochondrial reactive oxygen species (ROS)^60^ and glutathione levels^61^ have indeed been reported previously in rodent BAT and WAT. Browning of WAT has also been suggested to occur as an adaptive mechanism to alleviate redox pressure^62^. Increased expression of oxidative stress-related proteins has also been reported in eAT relative to sAT in patients undergoing cardiac surgeries^63^, an increase in ROS being further associated with reduced expression of *Ucp1* and related thermogenic markers in the eAT of patients with CAD^64^. A specific case for mitochondrial ROS and its regulation by mitochondrial uncoupling has also been made in the perivascular adipose tissue-mediated regulation of vascular function^65,66^.

In conclusion, we have shown that human eAT exhibits consistent presence of *Ucp1* in conjunction with the expression of majority of known beige markers. eAT is capable of upregulating *Ucp1* mRNA expression and its uncoupling respiration upon stimulation. While thermogenic phenotype negatively associates with the oxidative stress-related parameters at the gene expression level, sympathetic stimulation of eAT is associated with a down-regulation of proteins associated with cellular redox homeostasis. Indeed, we have provided evidence to indicate that the communication between eAT endothelial cells can be altered positively by its sympathetic stimulation. Overall, we have provided evidence that *Ucp1* within eAT is physiologically relevant to its own biology and plausibly to its communication with surrounding cells. Our observations open avenues for future research focused upon expanding our understanding of the thermogenic potential of *Ucp1* in eAT and its therapeutic targeting for modulation of the cardiovascular outcomes in humans.

### Limitations and strengths of the study

A primary limitation of the study is that it is conducted using samples that are derived from older patients with multiple metabolic abnormalities who came in for various cardiac surgeries. Sexual dimorphism was also observed in the expression of some genes in eAT, mAT and sAT depots (Supplementary Table S5b,c). Thus, we acknowledge that an uneven distribution of gender could have affected our observations. In addition, since the amount of eAT around human heart is highly variable and dissecting too much eAT is not ideal, our sample size was also small, which resulted in pooling of samples or limited ability of addressing multiple questions in a given sample. An ideal approach to addressing our hypothesis would have been to utilize an animal model. However, rodents such as mice that have been well characterized for brown fat biology do not possess eAT^67,68^. Even when fed high fat diet or made obese, the fat accumulation in rodents often only occurs outside the pericardium, which makes them an unsuitable model for eAT related studies. Nevertheless, with advances made in brown fat biology, differences in the mouse and human brown and beige fat are becoming increasingly apparent^20^. Thus, we believe that limitation of the current study also becomes its major strength as we have addressed all our questions using human samples. Moreover, eAT, mAT and sAT samples were obtained in a pair-wise manner, thus our work allows for a more direct depot-specific investigation that is usually difficult to execute in human studies. Finally, these observations have been made in immensely variable settings yet they are consistent with the available literature in the field providing significant support to our conclusions.

## Methods

### Study population and biopsy collection

Patients undergoing various heart surgeries (*e*.*g*. coronary artery bypass grafting and valve replacement) at the Institut universitaire de cardiologie et de pneumologie de Québec (IUCPQ) participated in the study with written informed consent. Institutional ethics committee of IUCPQ approved the study. Adipose tissue biopsies from three compartments, namely epicardial, mediastinal, and subcutaneous, were taken from the chest of each individual. Detailed description of biopsy collection and inclusion/exclusion criteria are given in the online supplement. Immediately after resection, biopsies were either snap frozen in liquid nitrogen for mRNA isolation or fixed in 4% paraformaldehyde for Ucp1-immunostaining (detailed in online supplement). Biopsies from some patients were kept in saline at 37 °C and were directly utilized for primary cell culture and explant culture as needed. Historic data on mean outdoor temperature and daylight period on the day of tissue collection in the Quebec City region were obtained from Environment Canada (http://climate.weather.gc.ca/historical_data) and National Research Council Canada websites (http://app.hia-iha.nrc-cnrc.gc.ca/cgi-bin/sun-soleil.pl), respectively. We confirm that all experiments were performed in accordance with relevant guidelines and regulations.

### Human primary adipocyte and explant culture

#### Primary cell culture

Roughly 100 mg of paired eAT, mAT and sAT biopsies were subjected to collagenase digestion followed by isolation and culture of stromovascular fraction (detailed in online supplement). Preadipocytes were differentiated using Preadipocyte Growth Media kit (Lonza, CA) as per manufacturer’s instructions. Differentiated adipocytes were subjected to downstream analyses on day 21, which included Oil red O staining, relative quantification of gene expression and/or OCR assays using Seahorse XF^e^ Bioanalyzer (Agilent).

#### Primary explant culture

50–100 mg of paired eAT, mAT and sAT biopsies were collected and excised into smaller sections of 10 mg in sterile and warm phosphate-buffered saline containing 1% penicillin/streptomycin (1% P/S) (kept at 37 °C) quickly while dissecting away any visible blood vessels. Sections were immediately transferred to fetal-bovine serum-free explant culture media (DMEM-F12 containing 33 μM biotin and 17 μM D-pantothenate and 1% P/S). Six hours later, explants were incubated with fetal-bovine serum-free explant culture media with or without 100 μM ISO in a 1: 10 ratio of tissue (mg)/media (μl)) at 37 °C. Twenty four hours later, explants were collected and flash frozen for mRNA isolation, whereas conditioned media was collected, filtered and flash-frozen for subsequent analyses.

#### Primary cardiac endothelial cell culture

Primary human cardiac endothelial cells were isolated from normal right ventricular microvessels of a 41 year-old women undergoing diagnostic cardiac surgery at IUCPQ. CD31^+^ cells were isolated using Dynabeads CD31^+^ selection (Invitrogen, CA) following the manufacturer’s instructions^69^. Cells were subsequently grown in Endothelial Basal Media-2 (EBM-2) (Lonza, CA) as per given instructions. 50,000 cells were plated in a 12-well plate a day prior to the experiment. Cells were incubated with 500 μl of CM with (Iso-CM) or without ISO (*i*.*e*. Ctl-CM) or diluted with EBM-2 media in 1:10 proportion for twenty-four hours, after which cells were washed with saline and immediately used for mRNA isolation.

### Oxygen consumption analyses

Primary preadipocytes were plated (20,000 cells/well) and differentiated in XF24 V7 PET cell culture microplates using the protocol described above. On the day of assay, cells were switched to XF-OCR assay media containing 2% free-fatty acid free BSA in the presence or absence of dibutyryl cAMP for four hours prior to the mito-stress assay (described in detail in the online supplement) using XFe24 Seahorse bioanalyzer (Agilent XF Seahorse).

For fatty acid oxidation (FAO) assay, cells were switched from growth media to substrate-limited media twenty four- hours prior to the assay. Forty five minutes prior to the assay, cells were switched to FAO media and incubated in the non-CO_2_ incubator at 37 °C. Etomoxir (40 μM) was added to the specified wells fifteen minutes prior to the assay. XF palmitate-BSA FAO (100 μM) substrate or BSA control were added right before running the mito-stress as per the protocol described in Rogers *et al*.^70^.

### Proteomic analysis of the conditioned media

Ctl and Iso-treated CM samples were analyzed by nanoLC/MSMS in triplicates for statistical information. For each injection, 1 μg of peptide sample was injected and separated by online reversed-phase (RP) nanoscale capillary liquid chromatography and analyzed by electrospray mass spectrometry. Further details on database search, label-free quantification and Metascape analyses of the data are given as part of the online supplement.

### Statistical Analysis

Data are expressed as mean ± SEM. Relative mRNA expression of individual gene was compared among eAT, mAT and sAT biopsies (n = 53) using randomized block ANOVA model in R. Missing data (when negligible level (*i*.*e*. CT values > 40) of expression was detected for a sample) were replaced by half of the lowest value in the respective gene category in order to perform various statistical analyses. Data were tested for normality and equality of variance and were log-transformed (following Box-Cox transformation). Parametric tests were used when normality criteria assessed using Shapiro-Wilk’s test was met in R, otherwise Friedman test followed by Dunn’s posthoc analysis was used to compare the groups using GraphPad Prism. Differences exhibiting a *P* ≤ 0.05 were considered significant.

For hierarchical cluster analyses, expression values for each gene in both eAT and mAT were first expressed as log fold-change relative to their values in sAT, respectively, followed by cluster analysis using the agglomerative WardD method with distance calculated using Manhattan criteria. In case of samples where non-detectable level of expression was observed (for *e*.*g*. *Ucp1* expression in sAT), an arbitrary CT value of 40 was assigned to derive fold change values. Heatmaps for the visualization of clustered data were generated using R.

Genes were grouped in various categories as the following: *Ucp1*, *Ppargc1a*, *Prdm16*, *Cpt1b*, *Cox4i1* as ‘thermogenesis’-related genes; *Tbx1*, *Tmem26*, *Tnfrsf9*, *P2rx5*, *Slc36a2* as ‘beige’-related genes’; *Shox2*, *Hoxc9*, *Slc7a10*, *Lep* as ‘white’-related genes; *Il6*, *Ccl2*, *Tnf*, *Ccl18*, *Mrc1*, *Cd68* as ‘immune’-related genes; *Col6a3*, *Col3a1*, *Mmp9*, *Timp1* as ‘extracellular matrix’-related genes; *Hif1a*, *Ncf1*, *Cyba*, *Tgfb1* as ‘oxidative stress’-related genes; *Slc2a4*, *Adipoq*, *Pparg*, *Fabp4*, *Cebpa* as the ‘adipose tissue growth and function’-related genes.

Genes within each group were compared among eAT, mAT and sAT using a multivariate randomized block ANOVA model, whereas, expression of genes within a group for a particular fat depot were compared among gender (males and females) or disease states (*i*.*e*. lean, overweight and obese individuals) or (CAD and non-CAD) or (diabetics and non-diabetics) using a multivariate ANOVA model using SAS studio. The relationships among various groups of genes within each fat depot were determined using the following method. First, all of the genes within a group (for a given fat depot) were subjected to principal component analysis and the first principal axis was retained in order to summarize the information about all of the genes as a one-dimension score. This score from each category was next used to assess the relations among various groups of genes using Pearson or Spearman correlation as well as stepwise regression using R. Relationships between *Ucp*1 in eAT and mean outdoor temperature and daylight on the day of tissue collection were determined by Pearson correlation analysis using GraphPad prism.

For the *in-vitro* studies, data were compared using unpaired t-tests or ANOVA (as specified in the figure legends) using GraphPad prism. Superscripts represent significant differences of *P* ≤ 0.05, unless otherwise specified.

### Data availability statement

The datasets generated during and/or analysed during the current study are available from the corresponding author on reasonable request.

## Electronic supplementary material

Supplementary information
