# Supplementary material for: Functional characterization of the Ucp1-associated oxidative phenotype of human epicardial adipose tissue
Source: Sci Rep. 2017 Nov 14;7:15566. doi: 10.1038/s41598-017-15501-7 (PMC5686183; doi:10.1038/s41598-017-15501-7)
Supplement: Supplementary file 1 — Supplementary information [file 41598_2017_15501_MOESM1_ESM.pdf]

Supplementary information for:

Functional characterization of the *Ucp1*-associated oxidative phenotype of human epicardial adipose tissue.

Kanta Chechi[1,2,8], Pierre Voisine[1,3,8], Patrick Mathieu[1,4,8], Mathieu Laplante[1,2,8], Sébastian Bonnet[1,5,8], Frédéric Picard[1,6,8] , Philippe Joubert[1,7,8] , Denis Richard[1,2.8*]

[1]Institut universitaire de cardiologie et de pneumologie de Québec (IUCPQ), 2725 Chemin Ste-Foy, Québec, G1V 4G5, Canada. [2]Department of Medicine, [3]Department of Cardiovascular Surgery, [4]Laboratory of Cardiovascular Pathobiology, Department of Surgery, [5]Pulmonary Hypertension and Vascular Biology Research Group, [6]Department of Pharmacy, [7]Department of Anatomo-Pathology, [8]Faculty of Medicine, Laval University, QC, Canada. Correspondence and requests for materials should be addressed to Denis Richard (email: Denis.Richard@criucpq.ulaval.ca).

Supplemental Methods

Study population and biopsy collection

70 patients undergoing coronary artery bypass grafting and valve replacement procedures between April 2014 and July 2016 at the IUCPQ were recruited for the study after obtaining written informed consent. Adipose tissue biopsies from three compartments, namely epicardial, mediastinal, and subcutaneous were taken from the chest of each individual during these procedures. eAT corresponds to the adipose depot in direct contact with the heart located between the myocardium and the visceral pericardium and was sampled from right atrial epicardium. mAT was sampled from the fat within the mediastinum, outside the pericardial sac. sAT was sampled from the anterior thorax after sternotomy.

Age, gender, anthropometric data including body surface area, body mass index, as well as systolic-, diastolic-blood pressure (SBP; DBP), clinical conditions (*i.e.* diabetes, hypertension, dyslipidemia and CAD), medication regime (use of statins, beta-blockers, calcium channel inhibitors and angiotensin converting enzyme- inhibitors) and biochemical data including plasma total-, low-density lipoprotein (LDL)-, high-density lipoprotein (HDL)-cholesterol and triglycerides were obtained from IUCPQ records collected prior to the surgical procedure and are given as Supplementary Tables 1a & b.

BMI was calculated as weight in kilograms divided by height in meters squared. Obesity, overweight, and normal weight were defined as BMI ≥ 30 kg/m^2, 25 to 29.9 kg/m^2, and 20 to 24.9 kg/m^2, respectively. Patients exhibiting impaired renal function, chronic inflammatory or autoimmune disease, cancer, and/or chronic obstructive pulmonary disease were excluded from the study.

Isolation and primary culture of human adipocytes

Roughly 100 mg of paired eAT, mAT and sAT biopsies were minced and subjected to digestion using DMEM-F12 (Life technologies) containing collagenase II (1.5 mg/ml) (Worthington type 2) and 3% fatty acid-free bovine serum albumin (Sigma Aldrich) for 30 min at 37°C with gentle shaking. The suspension was filtered through a cell strainer (100 micron size) followed by removal of floating mature adipocytes. Remaining suspension was centrifuged for 5 minutes at 800 g, pellet resuspended in DMEM-F12 and passed through another filter (30 micron). Finally, stromovascular fraction was suspended in DMEM-F12 containing 33μM biotin, 17μM D-pantothenate, 1% P/S and 10% FBS (Life technologies) and seeded in a 5 cm culture dish. Twenty-four hour later, media was switched to preadipocyte basal media (Lonza). Cells were grown until 80% confluency and were then split into a 10 cm dish (passage 1). Cells were either frozen after passage 3 or plated for mRNA isolation or OCR assays. Preadipocytes were

differentiated using Preadipocyte Growth Media kit (Lonza, CA) as per manufacturer's instructions until day 21 with additional presence of 1uM RSG (Cayman chemicals) and 2nM T3 (Sigma Aldrich).

RNA extraction, reverse transcription and Quantitative PCR

Total RNA was isolated from tissue or cells using the RNeasy Lipid Tissue Mini Kit (QIAGEN, Mississauga, Ontario) according to manufacturer's instructions. Purity of total RNA was determined as 260/280 nm absorbance ratio with expected values between 1.8-2.0 using Biodrop Duo (BioDrop, UK). In addition, RNA integrity of randomly selected samples (n =30, in tissue biopsies) was assessed using the Agilent 2100 Bioanalyzer (Agilent technologies, Santa Clara, California). 500ng of extracted total RNA was reverse-transcribed using iScript Advanced cDNA Synthesis kit (Bio-Rad Laboratories Ltd., Canada). The cDNA was diluted 1:20 in DNase-free water and was subjected to quantitative PCR using SsoAdvanced Universal SYBR Green Supermix (Bio-Rad Laboratories Ltd., Canada). The qPCR was performed in CFX384-Touch Real-Time PCR Detection System (Bio-Rad Laboratories Ltd., Canada). At the end of each run, melting curve analysis was performed to validate product specificity, and a few samples representative of each experimental group were run on agarose gel to verify specificity of the amplification followed by sequencing of the amplified product. All samples were amplified in duplicates and mean values were used for further analysis. Expression values were determined using standard curve method for each target and reference gene. A normalization factor calculated from the mean of expression levels of *Ppia*, *Gapdh* and *Rpl27* was used for the normalization process of the biopsies data (n=53), which was derived from the validation methods described previously[1,2]. All *in-vitro* data (adipocytes and endothelial cells) were normalized to *Gapdh* levels alone. Primer details are given as Supplementary Table 7.

Ucp1- immunohistochemical analysis

Ucp1-immunostaining was performed on sections of 5-µm-thick formalin-fixed paraffin-embedded adipose tissue samples using an automated immunohistochemistry protocol involving EnVision FLEX mini kit, high pH (Agilent technologies), with UCP1 antibody AC10983 (Abcam, USA) at 1:1000 dilution for use with Dako Autostainer 48 at the Department of Histopathology, IUCPQ.

Oil Red O staining

Cells were washed with PBS followed by incubation with 10% paraformaldehyde for 15 minutes to O/N. Cells were washed 3 times with PBS followed by 100% propylene glycol wash

followed by incubation with ORO stain (Sigma-Aldrich) at 37°C for 1 hour. Excess stain was discarded followed by addition of 85% propylene glycol. Cells were washed with water until all excess stain is removed. Pictures were taken at this step. ORO stain retained by adipocytes was next eluted using NP40 in isopropanol and quantified by measuring absorbance at 520nm.

Oxygen consumption analyses

Primary preadipocytes were plated (20,000 cells/well) and differentiated in XF24 V7 PET cell culture microplates using the protocol described above. On the day of assay, cells were switched to XF-OCR assay media (XF base medium + 1mM sodium pyruvate+ 2mM L-glutamine + 1mM HEPES + 2% free-fatty acid free BSA) in the presence or absence of dibutyryl cAMP (Db-cAMP) (1mM) for 4 hours prior to the assay. Mito-stress test using oligomycin (Tocris Biosciences)(5µM), FCCP (10µM) (Tocris Biosciences) and antimycin (5µM)/rotenone (3µM) (Cayman Chemicals) were then conducted using Seahorse XF^e Bioanalyzer as per manufacturer's instructions (Agilent). Post XF assay, cells were washed with PBS and fixed using 10% PFA followed by ORO staining. Mitochondrial respiration was calculated by subtracting non-mitochondrial respiration rates (determined as the respiration rate post antimycin/rotenone addition from OCR values. Basal respiration was calculated as the average of the values before the addition of oligomycin. Leak respiration was taken to be the lowest OCR value after the addition of oligomycin. Maximal respiration represented the highest OCR values after the addition of FCCP. Coupling efficiency was calculated as the ratio of basal respiration and ATP turnover (calculated as the difference of leak respiration from basal respiration).

For fatty acid oxidation assay, cells were switched from differentiation media to substrate-limited media (XF-base medium+ 0.5mM glucose+ 1mM L-glutamine + 0.5mM carnitine and 1% FBS) 24 hours prior to the assay. 45 minutes prior to the assay, cells were switched to FAO media (KHB+ 2.5mM glucose + 0.5mM carnitine + 5mM HEPES adjusted to pH 7.4 at 37°C) and incubated in the non-CO_2 incubator at 37°C. Etomoxir (40µM) (Sigma Aldrich) was added in the specified wells 15 minutes prior to assay and finally XF palmitate-BSA FAO (100µM) (Agilent XF Seahorse) substrate or BSA control were added before running the mito-stress test (as specified above) using XF^e24 Seahorse bioanalyzer as per the protocol.

LC-MS analyses of conditioned media

Mass spectrometry

Samples were analysed by nanoLC/MSMS as triplicates for statistical information. For each injection, 1µg of peptide samples were injected and separated by online reversed-phase (RP)

nanoscale capillary liquid chromatography (nanoLC) and analyzed by electrospray mass spectrometry (ESI MS/MS). The experiments were performed with a Dionex UltiMate 3000 nanoRSLC chromatography system (Thermo Fisher Scientific / Dionex Softron GmbH, Germering, Germany) connected to an Orbitrap Fusion mass spectrometer (Thermo Fisher Scientific, San Jose, CA,USA) driving with Orbitrap Fusion Tune Application 2.0 and equipped with a nanoelectrospray ion source. Peptides were trapped at 20 µl/min in loading solvent (2% acetonitrile, 0.05% TFA) on a 5mm x 300 µm C18 pepmap cartridge pre-column (Thermo Fisher Scientific / Dionex Softron GmbH, Germering, Germany) during 5 minutes. Then, the pre-column was switched online with a self-made 50 cm x 75µm internal diameter separation column packed with ReproSil-Pur *C18-AQ* 3-µm resin (Dr. Maisch HPLC GmbH, Ammerbuch-Entringen, Germany) and the peptides were eluted with a linear gradient from 5-40% solvent B (A: 0,1% formic acid, B: 80% acetonitrile, 0.1% formic acid) in 90 minutes, at 300 nL/min. Mass spectra were acquired using a data dependent acquisition mode using Thermo XCalibur software version 3.0.63. Full scan mass spectra (350 to 1800m/z) were acquired in the orbitrap using an AGC target of 4e5, a maximum injection time of 50 ms and a resolution of 120 000. Internal calibration using lock mass on the m/z 445.12003 siloxane ion was used. Each MS scan was followed by acquisition of fragmentation MSMS spectra of the most intense ions for a total cycle time of 3 seconds (top speed mode). The selected ions were isolated using the quadrupole analyzer in a window of 1.6 m/z and fragmented by Higher energy Collision-induced Dissociation (HCD) with 35% of collision energy. The resulting fragments were detected by the linear ion trap in rapid scan rate with an AGC target of 1e4 and a maximum injection time of 50 ms. Dynamic exclusion of previously fragmented peptides was set for a period of 20 sec and a tolerance of 10 ppm.

Database searching and Label Free Quantification

Spectra were searched against a human proteins database (Uniprot Complete Proteome – taxonomy Homo sapiens – 92042 sequences) using the Andromeda module of MaxQuant software v. 1.5.2.8 [3]. Trypsin/P enzyme parameter was selected with two possible missed cleavages. Carbamidomethylation of cysteins was set as variable modification, methionine oxidation and acetylation of protein N-terminus as variable modifications. Mass search tolerances were 5ppm and 0.6Da for MS and MS/MS, respectively. For protein validation, a maximum False Discovery Rate of 1% at peptide and protein level was used based on a target/decoy search. MaxQuant was also used for Label Free Quantification. The 'match between runs' option was used with 20 min value as alignment time window and 3 min as match time window. Only unique and razor peptides were used for quantification. The LFQ

intensity values (normalized values) extracted by MaxQuant for each protein in each sample replicate were used to calculate the ratio of intensities averages between the two samples to compare as well as a z-score calculated as follow: $z = (x-\mu)/\sigma$ where x = ratio of intensities averages, μ= median of all ratios of quantifiable proteins, σ = standard deviation of all ratios of quantifiable proteins. A p-value based on a Welch's *t*-test (modified Student's *t*-test) was also calculated. When LFQ intensity values were missing, there were replaced by a noise value corresponding to the first percentile of LFQ values of all proteins of the sample replicate. A protein was considered as quantifiable only if at least two replicate values in one of the two samples to compare were present. A protein was considered as variant if the absolute value of its z-score was higher than 1.96 and the associated p-value was lower than 0.05.

Metascape Analysis

Metascape first identified all statistically enriched terms (can be GO/KEGG terms, canonical pathways, hall mark gene sets, etc.), accumulative hypergeometric p-values and enrichment factors were calculated and used for filtering. Remaining significant terms were then hierarchically clustered into a tree based on Kappa-statistical similarities among their gene memberships. Then 0.3 kappa score was applied as the threshold to cast the tree into 'enrichment term' clusters.

Subsets of representative enrichment terms from cluster above are converted into a network layout. More specifically, each term is represented by a circle node, where its size is proportional to the number of input genes falling into that term, and its color representing its cluster identity (*i.e.*, nodes of the same color belong to the same cluster). Terms with a similarity score > 0.3 are linked by an edge (the thickness of the edge represents the similarity score). The network is visualized with Cytoscape (v3.1.2) with "force-directed" layout and with edge bundled for clarity. One term from each cluster is selected to have its term description shown as label.

Supplemental Figure legends:

Supplementary figure S1: Preliminary analysis of paired eAT-, mAT- and sAT-derived adipocytes. ORO staining (A) and OCR analyses (B) of eAT-, mAT- and sAT-derived adipocytes obtained from 4 patients. Images (A) represent the differentiation state on Day 21 for all cell types. SVF were plated after passage 2 and differentiated *in-vitro* using PGM-2 media containing 1uM RSG and 2nM T3. XF-mito-stress test was conducted on these adipocytes on day 21 in the presence of 2% BSA in the XF-assay media using 5μM oligomycin, 10μM FCCP and 5μM antimycin/3μM rotenone in Seahorse XF^e Bioanalyzer. ORO, oil red O; OCR, oxygen consumption rate; SVF, stromo-vascular fraction; PGM-2, preadipocyte growth media-2; RSG, rosiglitazone; BSA, bovine serum albumin.

Supplementary Figure S2: OCR analyses of the *in-vitro* differentiated eAT-, mAT- and sAT-derived adipocytes obtained after pooling samples from 9 patients. Data are expressed as mean ± SEM, n=10. Differences were estimated using one-way ANOVA followed by Tukey's *post hoc* analysis. Different alphabets represent $P \leq 0.05$. Maximal respiration represented the highest OCR values after the addition of FCCP. Spare respiratory capacity was calculated by subtracting the basal respiration rates from the maximal respiration states. ORO levels were used to normalize the OCR data. ORO, oil red O; OCR, oxygen consumption rate.

Supplementary Table S1: Clinical characteristics of the patients in the main cohort (n=53) (S1a). Clinical characteristics of the patients included in the *in-vitro* analyses (S1b).

Parameters	Value
Age (years)	64.57 ± 1.38
Body mass index (kg/m^2)	29.08 ± 0.82
Body surface area (m^2)	1.89 ± 0.03
Triglycerides (mM)	1.29 ± 0.08
Total-cholesterol (mM)	4.11 ± 0.20
LDL-cholesterol (mM)	2.26 ± 0.18
HDL-cholesterol (mM)	1.29 ± 0.06
Systolic blood pressure (mmHg)	133.36 ± 2.89
Diastolic blood pressure (mmHg)	71.63 ± 1.23
Sex (females/males)	17/36
Diabetes (Yes/No)	19/34
Heart surgeries (CABG/VR)	35/18
Hypertension (Yes/No)	15/38
Infarction (Yes/No)	13/40
Statins	42/11
ACE inhibitors	22/31
Ca channel inhibitors	18/35
Beta-blocker (Yes/No)	25/28

**Patients included in the preliminary seahorse analysis (n=4)**									
**Subjects**	**Surgery ***	**Gender**	**Age**	**BMI**	**BSA**	**Diabetes**	**Hypertension**	**Dyslipidemia**	**B-blocker**
Subject 1	CABG	♂	50	33.8	2.24	No	Yes	Yes	Yes
Subject 2	CABG	♂	57	22.7	1.86	No	Yes	Yes	Yes
Subject 3	CABG	♀	69	19.2	1.48	No	No	Yes	No
Subject 4	CABG	♀	78	30.5	1.82	No	Yes	Yes	Yes
**Patients included in the pooled cell culture (n=9)**									
Subject 5	VR_A+M	♀	77	20.7	1.51	No	Yes	No	No
Subject 6	VR_M	♀	75	18.8	1.35	No	Yes	Yes	Yes
Subject 7	VR_A+M	♀	70	28.2	1.77	No	No	Yes	No
Subject 8	VR_A	♂	51	31.9	2	No	Yes	Yes	Yes
Subject 9	VR_A	♂	72	31.3	2.04	No	Yes	Yes	No
Subject 10	VR_A	♀	68	39.5	1.96	Yes (type 2)	Yes	Yes	No
Subject 11	CABG	♂	64	23.8	1.93	No	Yes	Yes	Yes
Subject 12	CABG	♂	73	27.7	2.02	Yes	No	Yes	No
Subject 13	VR_A+M	♂	52	22.7	1.81	Yes (type 2)	No	Yes	No
**Patients included in the explant culture (n=4)**									
Subject 1	CABG	♂	57	31.9	2.29	No	Yes	Yes	Yes
Subject 2	VR_A+CABG	♂	68	29.9	1.98	No	Yes	Yes	Yes
Subject 3	VR_A+CABG	♂	67	38.4	1.81	No	Yes	Yes	Yes
Subject 4	VR_A+M	♀	68	24.8	1.71	Yes (type 2)	Yes	Yes	Yes

*CABG, coronary artery bypass grafting; VR_A, aortic valve replacement; VR_M, Mitral valve replacement; VR_A+M, aortic and mitral valve replacement. ACE inhibitors

Supplementary Table S2: Patients characteristics (S2a), expression of various groups of genes (S2b) and of individual genes (S2c) in lean (n =14), overweight (n =18) and obese (n = 21) patients in the main cohort (n=53).

Table S2a	Lean (n=14) (BMI ≤ 25)	Overweight (n=18) (BMI ≤ 25.1-29.9 ≥)	Obese (n=21) (BMI ≥ 30)
**Sex (female/male)**	7F/7M	1F/17M	9F/12M
**Age (years)**	63.9 ± 3.2	67.4 ± 1.9	62.6 ± 2.2
**Body weight (kg)**	61.2 ± 2.8[c]	80.8 ± 2.02[b]	94.5 ± 2.6[a]
**BMI (kg/m²)**	22.1 ± 0.55[c]	27.65 ± 0.33[b]	35.0 ± 0.83[a]
**BSA**	1.7 ± 0.05[b]	1.9 ± 0.03[a]	2.0 ± 0.04[a]
**Total-cholesterol (mM)**	4.1 ± 0.21	3.8 ± 0.25	4.3 ± 0.43
**LDL-cholesterol (mM)**	2.10 ± 0.18	2.18 ± 0.24	2.4 ± 0.39
**HDL-cholesterol (mM)**	1.53 ± 0.12[a]	1.17 ± 0.07[b]	1.23 ± 0.09[ab]
**Triglycerides (mM)**	1.03 ± 0.12	1.3 ± 0.13	1.46 ± 0.14

Table S2b	Lean (n=14) (BMI ≤ 25)	Overweight (n=18) (BMI ≤ 25.1-29.9 ≥)	Obese (n=21) (BMI ≥ 30)
eAT			
Thermogenesis	-1.25 ± 0.11	-1.21 ± 0.09	-1.21 ± 0.09
Beige	-0.29 ± 0.25	-0.81 ± 0.22	-0.36 ± 0.20
White	-1.06 ± 0.15	-1.25 ± 0.13	-1.01 ± 0.12
Inflammatory	-1.79± 0.14	-1.56 ± 0.12	-1.59 ± 0.11
Extracellular matrix	-1.02 ± 0.17	-1.02 ± 0.15	-0.72 ± 0.13
Oxidative stress	-1.38± 0.26	-1.36± 0.23	-0.97± 0.21
AT growth and function	0.87± 0.15	0.85 ± 0.13	1.06 ± 0.12
Adrenergic receptors	-0.82 ± 0.18	-0.83 ± 0.16	-0.59 ± 0.15
mAT			
Thermogenesis	-1.52± 0.19	-1.35 ± 0.17	-1.26 ± 0.15
Beige	-1.22 ± 0.36	-1.04 ± 0.31	-1.16 ± 0.29
White	-0.06 ± 0.18	0.04 ± 0.16	0.23 ± 0.15
Inflammatory	-1.84 ± 0.18	-1.50 ± 0.16	-1.84 ± 0.15
Extracellular matrix	-1.03 ± 0.17	-0.95 ± 0.15	-1.01 ± 0.14
Oxidative stress	-1.42± 0.22	-1.01± 0.19	-1.05± 0.18
AT growth and function	1.01 ± 0.20	0.67 ± 0.18	0.90 ± 0.17
Adrenergic receptors	-0.52 ± 0.21	-0.64 ± 0.19	-0.49 ± 0.17
sAT			
Thermogenesis	-2.31 ± 0.25	-2.40 ± 0.22	-2.46 ± 0.09
Beige	-2.00 ± 0.29	-1.96 ± 0.24	-2.39 ± 0.24
White	0.57 ± 0.17	0.29 ± 0.15	0.61 ± 0.14
Inflammatory	-1.21± 0.18	-1.13 ± 0.14	-0.99 ± 0.14
Extracellular matrix	-1.22 ± 0.21	-1.02 ± 0.18	-1.15 ± 0.17
Oxidative stress	-2.50± 0.25	-2.00± 0.22	-2.02± 0.20
AT growth and function	0.80 ± 0.17	0.55 ± 0.15	0.54 ± 0.14
Adrenergic receptors	-1.00 ± 0.22	-1.01 ± 0.20	-0.98 ± 0.18

Table S2c	Lean (n=14) (BMI ≤ 25)	Overweight (n=18) (BMI ≤ 25.1-29.9 ≥)	Obese (n=21) (BMI ≥ 30)
eAT			
Lep	0.598 ± 0.164[b]	0.900 ± 0.145[ab]	1.275 ± 0.134[a]
Slc36a2	0.317 ± 0.367[a]	-0.972 ± 0.323[b]	-0.307 ± 0.300[ab]
Il6	-4.871 ± 0.404[b]	-3.436 ± 0.356[a]	-4.590 ± 0.330[b]
Ccl2	0.424 ± 0.240[b]	1.263 ± 0.212[a]	0.761 ± 0.196[ab]
Col3a1	-1.373 ± 0.118[a]	-1.707 ± 0.104[b]	-1.385 ± 0.096[a]
Adra2a	0.217 ± 0.141[b]	0.423 ± 0.124[ab]	0.647 ± 0.115[a]
Adrb2/2a	-0.234 ± 0.252[a]	-0.944 ± 0.222[b]	-0.576 ± 0.206[ab]
mAT			
Lep	-0.015 ± 0.233[b]	0.496 ± 0.206[ab]	0.969 ± 0.191[a]
Il6	-4.504 ± 0.491[ab]	-3.763 ± 0.433[a]	-5.466 ± 0.401[b]
Mrc1	1.207 ± 0.176[b]	1.660 ± 0.157[ab]	1.803 ± 0.146[a]
Cyba	0.028 ± 0.200[b]	0.696 ± 0.177[a]	0.833 ± 0.164[a]
sAT			
Hoxc9	0.850 ± 0.344[a]	-0.252 ± 0.303[b]	0.604 ± 0.281[a]
Col3a1	-0.493 ± 0.133[a]	-0.880 ± 0.117[b]	-0.623 ± 0.109[ab]

Data are expressed as mean ± SEM, n= 53 (Table S2a) and as log of least square means ± SE, n= 53 (Table S2b & S2c). Data were compared using one-way ANOVA followed by Tukey's *post hoc* analysis (Table S2a). Genes were grouped in various categories as described in detail in the methods section and were compared using a multivariate ANOVA model (Table S2b). Superscripts represent significance of $P \leq 0.05$. * represents $P \leq 0.05$, ** represents $P \leq 0.01$, *** represents $P \leq 0.001$.

Supplementary Table 3: Patients characteristics (S3a), expression of various groups of genes (S3b) and of individual genes (S3c) in CAD and non-CAD patients in our cohort.

Table S3a	CAD (n=35)	NCAD (n=18)
**Sex (female/male)**	8F/27M	9F/9M
**Age (years)**	64.2 ± 1.4	65.2 ± 3.1
**Body weight (kg)**	80.9 ± 2.5	81.4 ± 4.9
**BMI (kg/m²)**	28.8 ± 0.97	29.6 ± 1.5
**BSA**	1.9 ± 0.03	1.9 ± 0.06
**Total-cholesterol (mM)**	4.05 ± 0.26	4.2 ± 0.30
**LDL-cholesterol (mM)**	2.2 ± 0.25	2.3 ± 0.23
**HDL-cholesterol (mM)**	1.26 ± 0.07	1.34 ± 0.10
**Triglycerides (mM)**	1.3 ± 0.10	1.2 ± 0.12

Table S3b	CAD (n=35)	NCAD (n=18)
eAT		
Thermogenesis	-1.26± 0.07	-1.13 ± 0.09
Beige	-0.65± 0.15$	-0.18 ± 0.21
White	-1.05± 0.09	-1.22 ± 0.13
Inflammatory	-1.61 ± 0.09	-1.67 ± 0.12
Extracellular matrix	-0.94 ± 0.21	-0.84 ± 0.15
Oxidative stress	-1.28 ± 0.16	-1.09 ± 0.23
AT growth and function	0.90 ± 0.09	1.00 ± 0.13
Adrenergic receptors	-0.74 ± 0.12	-0.71 ± 0.16
mAT		
Thermogenesis	-1.38± 0.12	-1.33 ± 0.17
Beige	-1.23 ± 0.22	-0.95 ± 0.31
White	0.05 ± 0.11	0.16 ± 0.16
Inflammatory	-1.51 ± 0.11a	-2.14 ± 0.15b
Extracellular matrix	-0.97 ± 0.11	-1.04 ± 0.15
Oxidative stress	-1.14 ± 0.14	-1.13 ± 0.19
AT growth and function	0.89 ± 0.13	0.78 ± 0.18
Adrenergic receptors	-0.48 ± 0.13	-0.68 ± 0.19
sAT		
Thermogenesis	-2.26 ± 0.15	-2.68 ± 0.21
Beige	-2.01 ± 0.18	-2.40 ± 0.25
White	0.42 ± 0.11	0.62 ± 0.15
Inflammatory	-1.09 ± 0.11	-1.10 ± 0.16
Extracellular matrix	-1.01 ± 0.13	-1.34 ± 0.18
Oxidative stress	-2.03 ± 0.16	-2.36 ± 0.22
AT growth and function	0.60 ± 0.11	0.64 ± 0.15
Adrenergic receptors	-0.90 ± 0.14	-1.18 ± 0.19

**Table S3c**	**CAD (n=35)**	**NCAD (n=18)**
eAT		
Tmem26	0.139 ± 0.138[b]	0.638 ± 0.193[a]
Timp1	-1.612 ± 0.129[b]	-1.081± 0.180[a]
mAT		
Il6	-4.111 ± 0.308[a]	-5.650 ± 0.429[b]
sAT		
Cpt1b	-1.441 ± 0.348[b]	-0.549 ± 0.250[a]
Prdm16	-2.563 ± 0.184[b]	-1.783 ± 0.132[a]
Fabp4	1.050 ± 0.130[b]	1.497 ± 0.181[a]

Data are expressed as mean ± SEM, n= 53 (Table S3a) and as log of least square means ± SE, n= 53 (Table S3b & S3c). Data were compared using unpaired t-tests (Table S3a). Genes were grouped in various categories as described in detail in the methods section and were compared using a multivariate ANOVA model (Table S3b, c). Superscripts represent significance of $P \leq 0.05$. "$" represents $P = 0.083$ (Table S3b).

Supplementary Table S4: Patients characteristics (S4a), expression of various groups of genes (S4b) and of individual genes (S4c) in diabetics (n=19) and non-diabetics (n= 34) in our cohort.

Table S4a	DM (n=19)	NDM (n=34)
Sex (female/male)	2F/17M	15F/19M
Age (years)	66.6 ± 2.1	63.4 ± 1.8
Body weight (kg)	91.1 ± 2.9	75.4 ± 2.8***
BMI (kg/m^2)	31.2 ± 1.1	27.9 ± 1.1*
BSA	2.0 ± 0.04	1.8 ± 0.4***
Total-cholesterol (mM)	3.6 ± 0.2	4.4 ± 0.29*
LDL-cholesterol (mM)	1.8 ± 0.12	2.5 ± 0.27*
HDL-cholesterol (mM)	1.1 ± 0.08	1.4 ± 0.07*
Triglycerides (mM)	1.5 ± 0.2	1.2 ± 0.07*

Table S4b	DM (n=19)	NDM (n=34)
eAT		
Thermogenesis	-1.21 ± 0.09	-1.22 ± 0.07
Beige	-0.57 ± 0.21	-0.44 ± 0.16
White	-1.05± 0.13	-1.14 ± 0.10
Inflammatory	-1.47 ± 0.11	-1.72 ± 0.09
Extracellular matrix	-0.72 ± 0.14	-1.00 ± 0.11
Oxidative stress	-1.06 ± 0.22	-1.30 ± 0.17
AT growth and function	0.97 ± 0.13	0.92 ± 0.09
Adrenergic receptors	-0.58 ± 0.15	-0.82 ± 0.12
mAT		
Thermogenesis	-1.54 ± 0.16	-1.26 ± 0.12
Beige	-1.67 ± 0.29[b]	-0.84 ± 0.22[a]
White	0.15 ± 0.16	0.06 ± 0.12
Inflammatory	-1.86 ± 0.16	-1.65 ± 0.12
Extracellular matrix	-0.98 ± 0.15	-1.01 ± 0.11
Oxidative stress	-1.23 ± 0.19	-1.08 ± 0.14
AT growth and function	0.78 ± 0.18	0.89 ± 0.13
Adrenergic receptors	-0.59 ± 0.18	-0.52 ± 0.14
sAT		
Thermogenesis	-2.43 ± 0.21	-2.38 ± 0.16
Beige	-2.39 ± 0.25	-2.00 ± 0.18
White	0.30 ± 0.14	0.60 ± 0.11
Inflammatory	-1.26 ± 0.15	-1.00 ± 0.11
Extracellular matrix	-1.02 ± 0.18	-1.18 ± 0.13
Oxidative stress	-2.04 ± 0.22	-2.19 ± 0.16
AT growth and function	0.44 ± 0.15	0.71 ± 0.11
Adrenergic receptors	-1.10 ± 0.19	-0.94 ± 0.14

**Table S4c**	**DM (n=19)**	**NDM (n=34)**
eAT		
Lep	1.234 ± 0.146[a]	0.821 ± 0.109[b]
Fabp4	0.278 ± 0.093[b]	0.522 ± 0.069[a]
Adra2a	0.674 ± 0.121[a]	0.336 ± 0.090[b]
mAT		
Slc36a2	-3.274 ± 0.523[b]	-1.587 ± 0.391[a]
Mrc1	1.947 ± 0.150[a]	1.401 ± 0.112[b]
Col3a1	-0.907 ± 0.143[a]	-1.270 ± 0.107[b]
sAT		
Tmem26	-1.882 ± 0.360[b]	-0.841 ± 0.269[a]
Adipoq	0.302 ± 0.146[b]	0.686 ± 0.109[a]

Data are expressed as mean ± SEM, n= 53 (Table S4a) and as log of least square means ± SE, n= 53 (Table S4b & S4c). Data were compared using unpaired t-tests (Table S4a). Genes were grouped in various categories as described in detail in the methods section and were compared using a multivariate ANOVA model (Table S4b, c). Superscripts represent significance of $P \leq 0.05$. * represents $P \leq 0.05$, ** represents $P \leq 0.01$, *** represents $P \leq 0.001$.

Supplementary Table S5: Patients characteristics (S5a), expression of various groups of genes (S5b) and of individual genes (S5c) in males (n=36) and females (n= 17) in our cohort.

Table S4a	Males (n=36)	Females (n=17)
**Age (years)**	63.8 ± 1.7	66.2 ± 2.3
**Body weight (kg)**	83.9 ± 2.2	75.1 ± 5.4
**BMI (kg/m²)**	28.4 ± 0.7	30.5 ± 2.1
**BSA**	2.0 ± 0.0	1.7 ± 0.1***
**Total-cholesterol (mM)**	3.8 ± 0.1	4.8 ± 0.5
**LDL-cholesterol (mM)**	2.1 ± 0.1	2.6 ± 0.5
**HDL-cholesterol (mM)**	1.2 ± 0.1	1.5 ± 0.1***
**Triglycerides (mM)**	1.3 ± 0.1	1.3 ± 0.1

Table S4b	Males (n=36)	Females (n=17)
eAT		
Thermogenesis	-1.33 ± 0.09	-1.33 ± 0.09
Beige	-0.68 ± 0.19	-0.62 ± 0.28
White	-1.57± 0.16	-1.53 ± 0.23
Inflammatory	-1.62 ± 0.08[a]	-1.72 ± 0.12[b]
Extracellular matrix	-0.96 ± 0.12	-1.05 ± 0.18
Oxidative stress	-1.28 ± 0.18	-1.32 ± 0.27
AT growth and function	0.92 ± 0.09	0.90 ± 0.14
Adrenergic receptors	-1.18 ± 0.14	-1.15 ± 0.20
mAT		
Thermogenesis	-1.46 ± 0.15	-1.54 ± 0.22
Beige	-1.69 ± 0.29	-1.40 ± 0.42
White	0.12 ± 0.14	-0.14 ± 0.20
Inflammatory	-1.64 ± 0.11	-1.95 ± 0.16
Extracellular matrix	-1.08 ± 0.12	-1.10 ± 0.18
Oxidative stress	-1.17 ± 0.16	-1.30 ± 0.23
AT growth and function	0.86 ± 0.12	0.76 ± 0.17
Adrenergic receptors	-1.24 ± 0.14[a]	-1.32 ± 0.20[b]
sAT		
Thermogenesis	-3.30 ± 0.25	-2.68 ± 0.37
Beige	-3.50 ± 0.32	-2.55 ± 0.47
White	0.32 ± 0.10[b]	0.77 ± 0.15[a]
Inflammatory	-2.80 ± 0.17	-2.57 ± 0.24
Extracellular matrix	-1.25 ± 0.17	-1.34 ± 0.25
Oxidative stress	-2.37 ± 0.19	-2.17 ± 0.28
AT growth and function	0.46 ± 0.10[b]	0.75 ± 0.15[a]
Adrenergic receptors	-1.93 ± 0.12	-1.66 ± 0.18

Table S4c	Males (n=36)	Females (n=17)
eAT		
Il6	-4.00 ± 0.25[a]	-4.93 ± 0.36[b]
mAT		
Adra2a	1.19 ± 0.14[a]	0.58 ± 0.20[b]
Lep	0.71 ± 0.16[a]	0.07 ± 0.23[b]
sAT		
Slc7a10	0.47 ± 0.16[b]	1.43 ± 0.23[a]
Tmem26	-3.21 ± 0.57[b]	-0.10 ± 0.83[a]
Slc2a4	-0.66 ± 0.16[b]	0.27 ± 0.24[a]
Adrb3	-6.05 ± 0.29[b]	-5.04 ± 0.43[a]

Data are expressed as mean ± SEM, n= 53 (Table S5a) and as log of least square means ± SE, n= 53 (Table S5b & S5c). Data were compared using unpaired t-tests (Table S5a). Genes were grouped in various categories as described in detail in the methods section and were compared using a multivariate ANOVA model (Table S5b, 5c). Superscripts represent significance of $P \leq 0.05$. * represents $P \leq 0.05$, ** represents $P \leq 0.01$, *** represents $P \leq 0.001$.

Supplementary Table S6: Detailed description of 48 proteins that were differentially expressed in ISO-stimulated eAT explant derived conditioned media relative to controls identified by LC-MS analysis.

Protein names	Gene names	Ctl vs Iso Welch's test	Ctl vs Iso Z-score
Selenoprotein M	SELM	0.040	-4.41
Mycophenolic acid acyl-glucuronide esterase, mitochondrial	ABHD10	0.014	-4.18
Protein canopy homolog 3	CNPY3	0.004	-3.72
Marginal zone B- and B1-cell-specific protein	MZB1	0.000	-3.69
Carboxylic ester hydrolase;Cocaine esterase	CES2	0.001	-3.52
Valacyclovir hydrolase	BPHL	0.004	-3.49
Reticulocalbin-2	RCN2	0.007	-3.42
Neudesin	NENF	0.004	-3.38
Protein-tyrosine-phosphatase;Receptor-type tyrosine-protein phosphatase C	PTPRC	0.014	-3.25
Complement component 1 Q subcomponent-binding protein, mitochondrial	C1QBP	0.002	-3.25
Thiosulfate sulfurtransferase	TST	0.010	-3.06
UDP-glucose:glycoprotein glucosyltransferase 1	UGGT1	0.021	-2.92
GDP-fucose protein O-fucosyltransferase 1	POFUT1	0.020	-2.88
KDEL motif-containing protein 2	KDELC2	0.003	-2.72
Deoxyuridine 5-triphosphate nucleotidohydrolase, mitochondrial	DUT	0.004	-2.67
GDH/6PGL endoplasmic bifunctional protein;Glucose 1-dehydrogenase;6-phosphogluconolactonase	H6PD	0.002	-2.55
Pleiotrophin	PTN	0.009	-2.55
Delta(3,5)-Delta(2,4)-dienoyl-CoA isomerase, mitochondrial	ECH1	0.004	-2.53
ES1 protein homolog, mitochondrial	C21orf33	0.000	-2.48
Endoplasmic reticulum resident protein 29	ERP29	0.010	-2.48
LDLR chaperone MESD	MESDC2	0.029	-2.45
Thioredoxin domain-containing protein 5	TXNDC5	0.000	-2.43
Malate dehydrogenase, mitochondrial;Malate dehydrogenase	MDH2	0.000	-2.41
Glycine amidinotransferase, mitochondrial	GATM	0.011	-2.34
Succinyl-CoA:3-ketoacid coenzyme A transferase 1, mitochondrial	OXCT1	0.015	-2.33
Sulfatase-modifying factor 2	SUMF2	0.014	-2.27
Protein canopy homolog 2	CNPY2	0.000	-2.27
Peroxiredoxin-4	PRDX4	0.001	-2.25
Chitinase-3-like protein 1	CHI3L1	0.000	-2.21
Liver carboxylesterase 1	CES1	0.002	-2.19
Migration and invasion enhancer 1	MIEN1	0.009	-2.18
Mesencephalic astrocyte-derived neurotrophic factor	MANF	0.000	-2.14
Cathelicidin antimicrobial peptide;Antibacterial protein FALL-39;Antibacterial protein LL-37	CAMP	0.003	-2.12
Endoplasmic reticulum aminopeptidase 1	ERAP1	0.003	-2.10
Protein disulfide-isomerase A6	PDIA6	0.001	-2.09
Prosaposin;Saposin-A;Saposin-B-Val;Saposin-B;Saposin-C;Saposin-D	PSAP	0.004	-2.09
Folate receptor beta	FOLR2	0.034	-2.08
Calsequestrin-2	CASQ2	0.005	-2.08
Citrate synthase;Citrate synthase, mitochondrial	CS	0.000	-2.05
Thioredoxin domain-containing protein 12	TXNDC12	0.000	-2.03
Protein disulfide-isomerase A4	PDIA4	0.001	-2.02
Endoplasmin	HSP90B1	0.001	-1.98
Protein disulfide-isomerase	P4HB	0.000	-1.97
Serum amyloid A protein;Serum amyloid A-4 protein	SAA2-SAA4;SAA4	0.027	2.16
Stromal interaction molecule 1	STIM1	0.039	2.25
Desmin	DES	0.016	2.69
Apolipoprotein B-100;Apolipoprotein B-48	APOB	0.019	2.97
Inhibin beta A chain	INHBA	0.011	4.09

Supplementary Table S7: Detailed description of the enrichment terms identified by Metascape analysis of 48 secreted proteins in Iso-stimulated eAT explant relative to control.

Category	Term	Description	LogP	Log(q-value)	InTerm_InList	Symbols
GO Biological Processes	GO:0045454	cell redox homeostasis	-9.628462017	-5.392	7/76	CAMP,P4HB,PDIA4,PDIA6,PRDX4,TXNDC12, TXNDC5
GO Biological Processes	GO:0006457	protein folding	-9.074728053	-5.139	9/222	P4HB,HSP90B1,PDIA4,PDIA6,PRDX4,ERP29, MESDC2,UGGT1,TXNDC5
Reactome Gene Sets	R-HSA-2173782	Binding and Uptake of Ligands by Scavenger Receptors	-3.985715691	-0.594	3/43	APOB,SAA1,HSP90B1,CNPY3,FOLR2,PDIA6,TXNDC5,C1QBP,PTN,PTPRC
GO Biological Processes	GO:0044712	single-organism catabolic process	-3.552304101	-0.219	8/814	APOB,DUT,ECH1,OXCT1,HSP90B1,TST,ABHD10,UGGT1
GO Biological Processes	GO:0005975	carbohydrate metabolic process	-3.036424761	0.000	7/746	CHI3L1,CS,MDH2,H6PD,POFUT1,ABHD10,UGGT1
Reactome Gene Sets	R-HSA-211859	Biological oxidations	-3.011481564	0.000	4/208	BPHL,CES1,CES2,ABHD10
GO Biological Processes	GO:0006874	cellular calcium ion homeostasis	-2.981867615	0.000	5/367	CASQ2,PTPRC,SAA1,STIM1,HSP90B1
GO Biological Processes	GO:0050864	regulation of B cell activation	-2.678139213	0.000	3/120	INHBA,PTPRC,MZB1
GO Biological Processes	GO:0001934	positive regulation of protein phosphorylation	-2.619653706	0.000	7/882	CHI3L1,INHBA,PSAP,PTPRC,SAA1,ERP29,NENF
GO Biological Processes	GO:2000241	regulation of reproductive process	-2.608155025	0.000	3/127	C1QBP,INHBA,PRDX4
GO Biological Processes	GO:1901701	cellular response to oxygen-containing compound	-2.543732001	0.000	7/910	FOLR2,INHBA,OXCT1,PSAP,PTN,HSP90B1,MZB1
GO Biological Processes	GO:0048514	blood vessel morphogenesis	-2.458017528	0.000	5/484	APOB,CHI3L1,PTN,POFUT1,ERAP1
Reactome Gene Sets	R-HSA-6798695	Neutrophil degranulation	-2.454199464	0.000	5/485	CHI3L1,PSAP,PTPRC,PRDX4,TXNDC5
GO Biological Processes	GO:2001242	regulation of intrinsic apoptotic signaling pathway	-2.445707943	0.000	3/145	P4HB,ERP29,TXNDC12
GO Biological Processes	GO:0002221	pattern recognition receptor signaling pathway	-2.349100387	0.000	3/157	C1QBP,HSP90B1,CNPY3

Supplementary Table S8: Details of the primer sequences used in the study.

Gene	Forward primer (5′-3′)	Reverse Primer (5′-3′)	Accession number
Ucp1	CTCTCAGGATCGGCCTCTAC	GAGTAGTCCCTTTCCAAAGACC	NM_021833
Ppargc1a	GGGATGATGGAGACAGCTATG	ATACTTGCTCTTGGTGGAAGC	NM_013261
Prdm16	CAGCACGGTGAAGCCTTTC	GCGTGCATCCGCTTGTG	NM_022114
Cpt1b	CTCTTCCAGAAGGCTGCTAAG	CATCTGCTACAGGGCCAAAG	NM_004377
Cox4i1	GCAGAAGCACTATGTGTACGG	CCAGTAAATAGGCATGGAGTTG	NM_001861
Tbx1	ACGACAACGGCCACATTATTC	CCTCGGCATATTTCTCGCTATCT	NM_005992
Tmem26	TGACCTGGCAGTACAGAACG	ACGCTGATTCCGATGTTCCA	NM_178505
Tnfrsf9	AGCTGTTACAACATAGTAGCCAC	TCCTGCAATGATCTTGTCCTCT	NM_001561
Slc36a2	CCTGCCACTGTATGCACATC	TAGTCCATGCATCACCGTGT	NM_181776
P2rx5	ATGTCGCCGACTACGTCATT	GTTGGGGGTCACAATCAGGTT	NM_002561
Slc7a10	CCCTCGAAGCCACAATGAGAT	CAGGGCAGTGCTAAGACAGG	NM_019849
Shox2	GGCCCGAGTGCAGGTTTGGT	AACTGGCTGGCGGCCCCTAT	NM_003030
Hoxc9	GCAGCAAGCACAAAGAGGAGAAG	GCGTCTGGTACTTGGTGTAGGG	NM_006897
Lep	TCTATGTCCAAGCTGTGC	TTGGAGGAGACTGACTGC	NM_000230
Mtus1	ATCTCAAGGCAGCTTTCCACG	TCGCTTGTTGACTTTCGACTC	NM_020749
Kcnk3	CTACGAGCACTGGACCTTCTT	CGTAAGGATGTAGACGAAGCTGA	NM_002246
Il6	GGATTCAATGAGGAGACTTGCC	TGGCATTTGTGGTTGGGTCA	NM_000600
Tnf	GCTGCACTTTGGAGTGATCG	GCTTGAGGGTTTGCTACAACA	NM_000594
Ccl2	CTCGCTCAGCCAGATGCAAT	TTGGGTTTGCTTGTCCAGGT	NM_002982
Mrc1	GCCTCGTTGTTTTGCGTCTT	TTGCCTGGTGTCCAGTAGGA	NM_002438
Ccl18	AAGAGCTCTGCTGCCTCGTCTA	CCCTCAGGCATTCAGCTTCA	NM_002988
Cd68	GTTGAGCAACTGGTGCAGAC	TCCCCTGGGCTGCCAGTA	NM_001251
Tgfb1	CGACTCGCCAGAGTGGTTAT	CAATTTCCCCTCCACGGCT	NM_000660
Hif1a	GTCTGAGGGGACAGGAGGAT	CTCCTCAGGTGGCTTGTCAG	NM_001530
Cyba	CGAGCGGCATCTACCTACTG	GCTTGATGGTGCCTCCGAT	NM_000101
Ncf1	GACTTCCTCTTTCCAGTGCATTT	CAGGTCCTGCCATTTCACCA	NM_000265
Col6a3	AAGCTCTTAGCCAGCACTCG	CACTTTACTGGGGCCGATGT	NM_004369
Col3a1	TGGAGGATGGTTGCACGAAA	ACAGCCTTGCGTGTTCGATA	NM_000090
Timp1	GAGAGACACCAGAGAACCCAC	CCACAGCAACAACAGGATGC	NM_003254
Mmp9	CCCCAGCGAGAGACTCTACA	AGAAGCCGAAGAGCTTGTCC	NM_004994
Slc2a4	GGTTCTTTCATCTTCGCCGC	TCCCCATCTTCGGAGCCTAT	NM_001042
Cebpa	GAGGGGAGAATTCTTGGGGC	CATTGGAGCGGTGAGTTTGC	NM_004364
Pparg2	TTGGGCTCCATAAAGTCACC	GCTGTGCAGGAGATCACAGA	NM_138712
Fabp4	AAGAGAAAACGAGAGGATGA	CACAGAATGTTGTAGAGTTCA	NM_001442
Adipoq	TTCCATACCAGAGGGGCTCA	GAGTCGTGGTTTCCTGGTCA	NM_004797
Adrb1	AGGGGAACGAGGAGATCTGT	CAGACGAGGATTGTGGGCTT	NM_000684
Adrb2	GGGTCTTTCAGGAGGCCAAA	ATGCCTAACGTCTTGAGGGC	NM_000024
Adra2a	ATCCTGGCCTTGGGAGAGAT	TCTCAAAGCAGGTCCGTGTC	NM_000681
Adrb3	TACTCTGCGCTGGCTTTTGA	AAAGGCTCAAGCTCACTCCC	NM_000025
Elovl3	TCGAGGAGTATTGGGCAACC	AATGCCCCACATCCTCACTG	NM_152310
Acadm	GCCATTGATGTGTGCTTATTG	GCCCATGTTTAATTCCTTTCTC	NM_000016
Acadl	AGGATTTATCAAGGGACGAAAG	TGTTGCACTGTCTGTAGGTGAG	NM_001608
Acadvl	TCGAGGGCCTCAAGATCC	GCTTGAGGGAAGGCACATAAC	NM_000018
Acaca	CGTTGCCACCCTGAGGTCTTT	GGTTCAGCTCCAGAGGTTGG	NM_198836
Acox1	GCCGCCGAGAGATCGAGAAC	GCAGGAACATGCCCAAGTGA	NM_004035
Vcam1	GGACCACATCTACGCTGACA	TTGACTGTGATCGGCTTCCC	NM_001078
Icam1	AGCTTCGTGTCCTGTATGGC	TTTTCTGGCCACGTCCAGTT	NM_000201
Gapdh	ATGTTCGTCATGGGTGTGAA	GGTGCTAAGCAGTTGGTGGT	NM_002046
Ppia	ATCCTAGAGGTGGCGGATTT	CACTCAGGTCTGAGCCACAA	NM_021130
Rpl27	GTGAAAGTGTATAACTACAATCACC	TCAAACTTGACCTTGGCCT	NM_000988

Supplemental references

1. Iacobellis, G. *et al.* Echocardiographic Epicardial Adipose Tissue Is Related to Anthropometric and Clinical Parameters of Metabolic Syndrome: A New Indicator of Cardiovascular Risk. *J. Clin. Endocrinol. Metab.* **88,** 5163–5168 (2003).
2. Payne, G. A., Kohr, M. C. & Tune, J. D. Epicardial perivascular adipose tissue as a therapeutic target in obesity-related coronary artery disease. *Br. J. Pharmacol.* **165,** 659–669 (2012).
3. Cox, J. & Mann, M. MaxQuant enables high peptide identification rates, individualized p.p.b.-range mass accuracies and proteome-wide protein quantification. *Nat. Biotechnol.* **26,** 1367–1372 (2008).

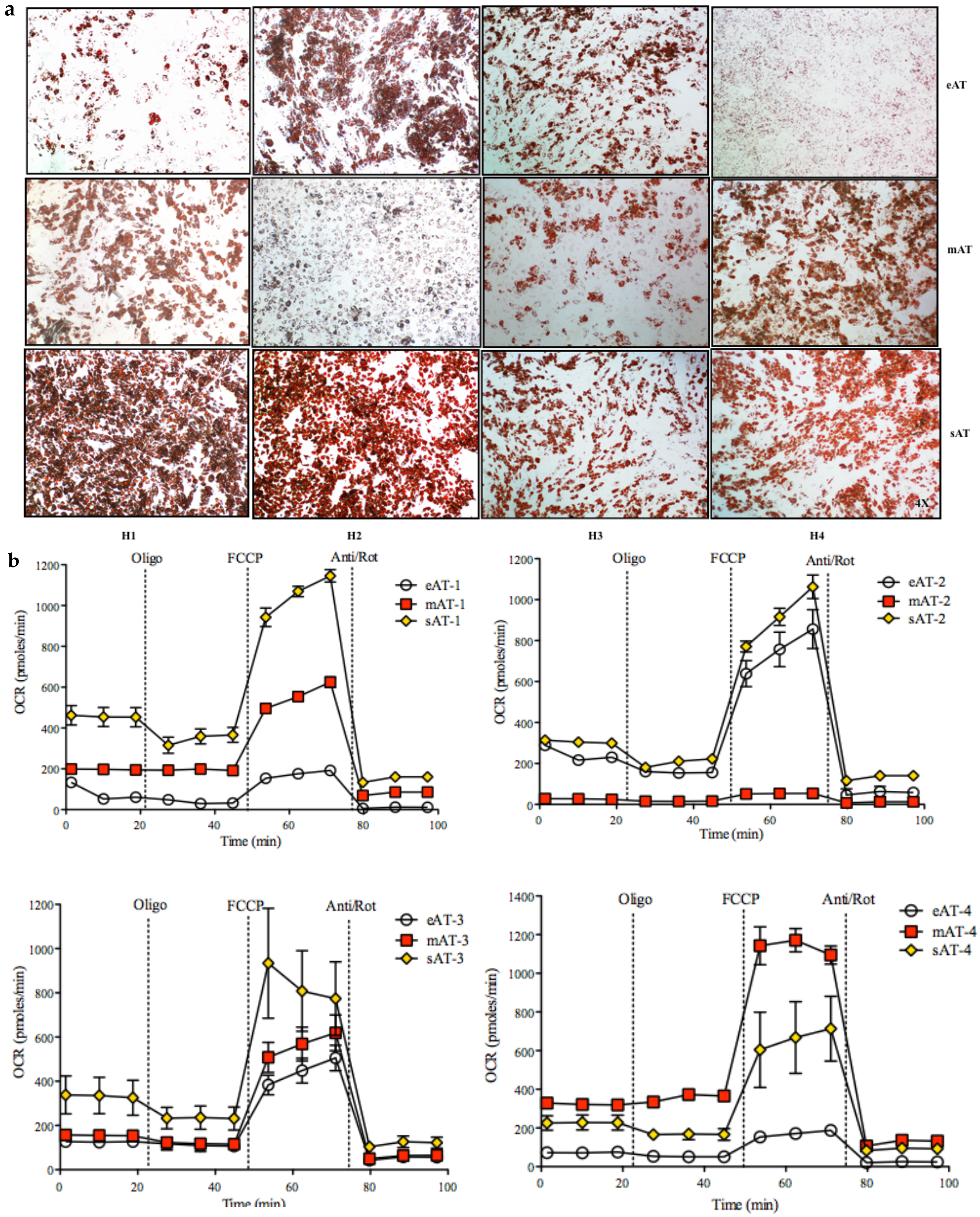

Supplementary figure S1: Preliminary analysis of paired eAT-, mAT- and sAT-derived adipocytes.

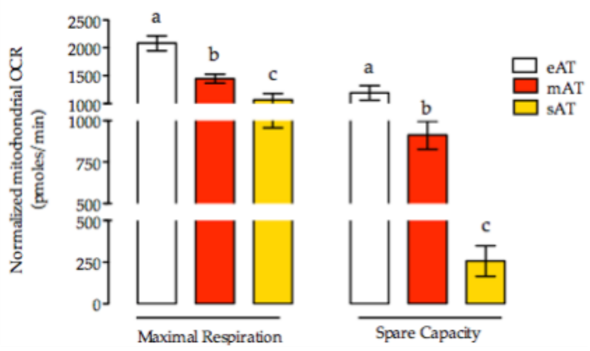

Supplementary Figure S2: OCR analyses of the *in-vitro* differentiated eAT-, mAT- and sAT-derived adipocytes obtained after pooling samples from 9 patients.
